# Light-Driven Lithium Extraction from Mixtures of Alkali Cations Using an Azobipyridine Ligand

**DOI:** 10.1021/jacs.5c05885

**Published:** 2025-06-09

**Authors:** Yuyin Du, Amit Ghosh, Paula C. P. Teeuwen, David J. Wales, Jonathan R. Nitschke

**Affiliations:** Yusuf Hamied Department of Chemistry, 2152 University of Cambridge, Cambridge CB2 1EW, United Kingdom

## Abstract

Humanity’s replacement of fossil-fuel energy with renewable electricity will require extensive deployment of lithium batteries, which necessitates an increase in the rate of production of this vital metal. Current extraction methods from brines and ores are very energy-intensive, as are recycling methods. Here we report a novel Li_5_ **L**_2_ sandwich structure in which five lithium ions are bound between the azobipyridine groups of two pentagonal ligands. The geometry of these ligands leads to the selectivity for binding Li^+^ in the presence of Na^+^ and K^+^. Upon illumination, the *trans*-azobipyridine moieties of the ligands isomerize to their *cis* form, promoting the release of free Li^+^ as the sandwich comes apart. The selective complexation of Li^+^, and its photostimulated release, were used as the basis of a cycle for selectively extracting and releasing Li^+^ ions from a mixture with Na^+^ and K^+^. Solar energy may thus be used directly to purify this metal, which is essential for storing increasingly cheap electricity produced through renewable means.

## Graphical abstract

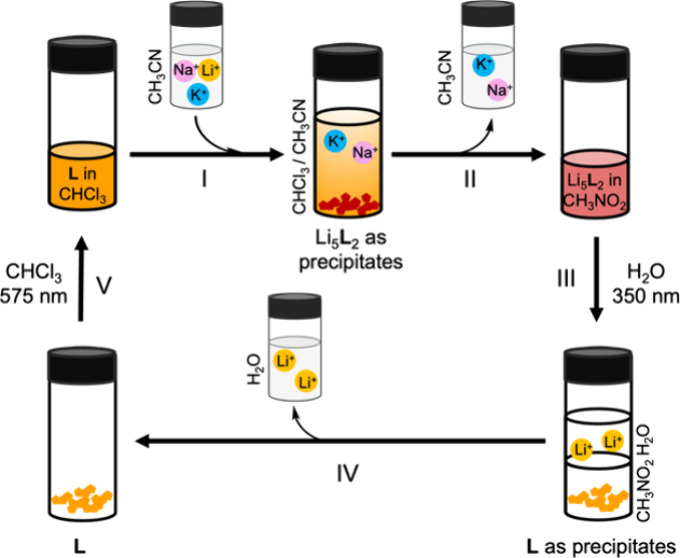

Lithium, the lightest metal, is in high demand due to its extensive applications in glass, ceramics, and especially lithium-ion batteries.^1–3^ Despite its technological relevance, the supply of lithium from brines and ores remains constrained.^4,5^ Over the past few decades, significant research efforts have been dedicated to developing more efficient lithium extraction methods. For example, solar evaporation and phosphate precipitation have been used to extract lithium from brines. However, these methods require highly concentrated brine and relatively long processing times.^6^ Lithium extraction using adsorbents, including metal–organic framework-based membranes, is an alternative method, although lower selectivity for Li^+^ over other metal ions reduces the overall efficiency.^7–10^ New and more efficient lithium purification methods are required to produce the vast amounts of lithium needed for batteries to drive the energy transition: these batteries are required to store electrical power from intermittent renewable sources.

Metal coordination can bring ligands that contain multiple binding sites together into complex and useful discrete structures.^11–17^ Many of these structures possess internal cavities capable of encapsulating guest molecules, enabling them to be used for chemical separation,^18–21^ catalysis,^22–26^ or biomedical applications.^27^ Building upon the use of crown ethers and cryptands to bind selectively to alkali metals,^28–31^ our group has recently demonstrated the first use of alkali metals to template the formation of iminopyridine-containing cages, by incorporating amines and aldehydes that are both tritopic. The resulting cages thus incorporate a single organic ligand that bridges between four metal ions, with the resultant high density of connections overcoming the low strength of coordination bonds to these metal ions.^32^

We envisaged that ligands incorporating robust, hydrolytically insensitive azo groups might also form stable metal–organic structures with alkali metals. These ligands possess useful redox-active properties and photoswitching behavior, potentially endowing their complexes with useful functions. Complementing iminopyridine-based structures, which have been shown to respond to chemical stimuli that include pH,^33–35^ concentration,^36–38^ or postassembly modifications^39–41^ to induce structural changes, photoresponsive azo-containing architectures can reconfigure when light is employed as a stimulus, offering a clean and cheap method without generating chemical waste. Our group has previously developed a Zn_4_L_4_ tetrahedral cage functionalized with azobenzene groups at its vertices, which undergoes reversible guest release and uptake upon photoisomerization, enabling the separation of steroids.^42,43^ While metal–organic cages incorporating azo moieties have demonstrated promise in various guest release applications,^44–48^ their use in metal extraction remains underexplored. Given that photoswitchable units such as azobenzene can modulate binding affinities upon isomerization,^49,50^ we hypothesized that incorporating azo groups into metal–organic complexes could allow light-controlled metal ion binding and release.

Here we present a Li_5_ **L**_2_ metal–organic sandwich structure **1** that incorporates pentatopic pyrrole-based ligand **L** (Figure 1A), as the use of pentatopic building blocks has garnered increasing attention for the construction of advanced supramolecular architectures and this ligand is synthetically feasible.^51–53^ Remarkably, this Li_5_ **L**_2_ complex formed selectively when **L** was treated with a mixture of Li^+^, Na^+^, and K^+^, due to the significantly lower binding affinity of **L** toward Na^+^ and K^+^. The *trans* to *cis* photoisomerization of the azo linkages within **L** allowed light to govern disassembly of the sandwich structure, thereby facilitating the release and subsequent extraction of Li^+^. Irradiation at a longer wavelength converted the *cis* linkages back to their *trans* form, recycling the ligand for further use. We were able to use this combination of selective Li^+^ binding and photostimulated release to design a cyclic process for the extraction of Li^+^ from other alkali metal cations. The iminopyridine-based analogs also showed selectivity toward Li^+^ (Scheme S6, Figures S23 and S24), but we did not pursue their use in extraction processes due to the absence of photoresponsive behavior.

**Figure 1:**
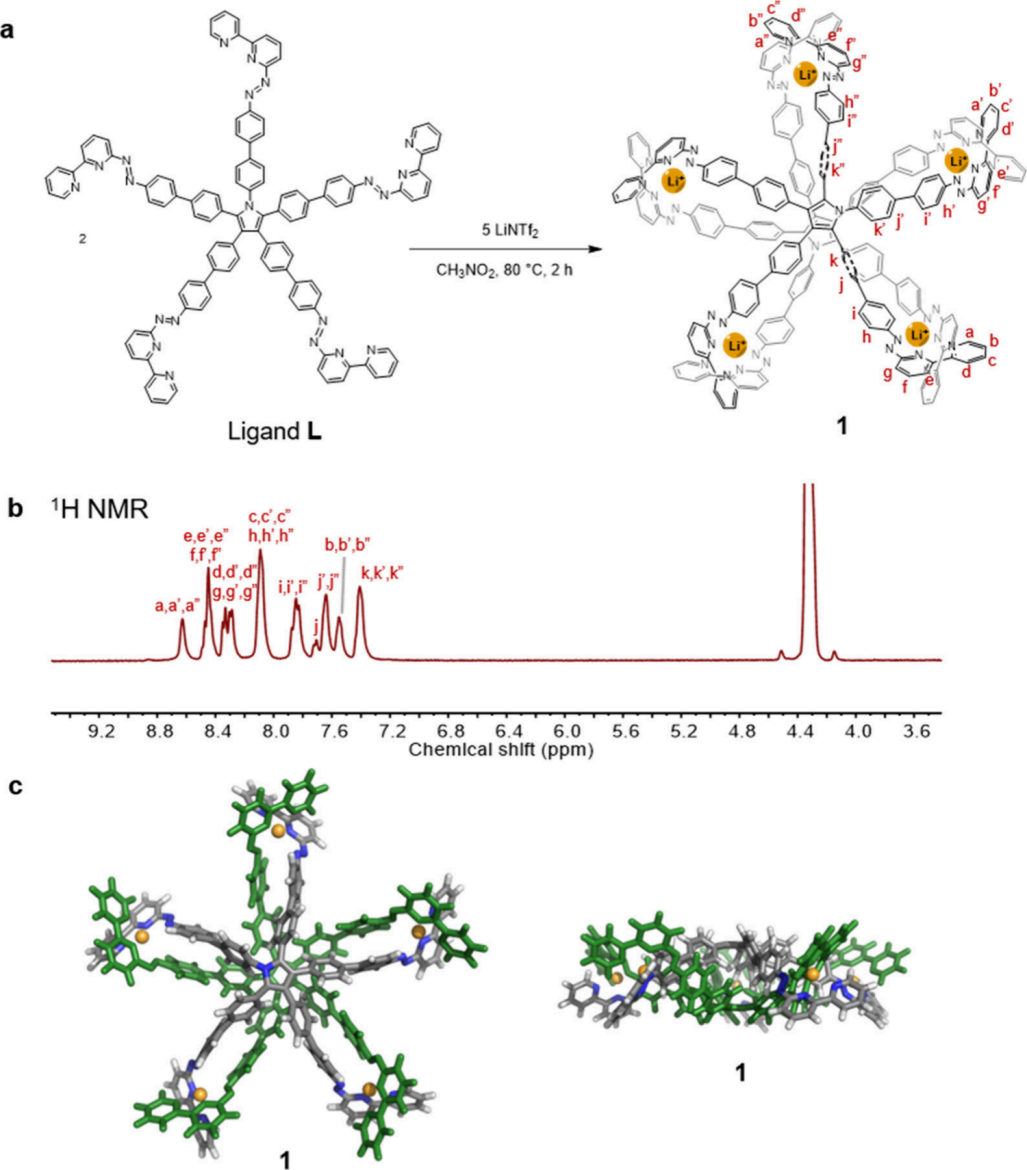
Self-assembly and characterization of Li_5_ **L**_2_sandwich structure **1**. (a) Self-assembly of complex **1**. (b) ^1^H NMR (400 MHz, CD_3_NO_2_, 363 K) spectra of **1** with peak assignments. (c) DFT minimized structure of **1** at the r^2^SCAN-3c level of theory, top and side views (Color codes: C, gray; N, blue; Li, yellow; H, white, with one ligand, **L**, shown in green for clarity).

Ligand **L** was synthesized as shown in Scheme S1. As illustrated in Figure 1a, **L** (1 equiv) reacted with lithium trifluoromethanesulfonimide (triflimide or ^–^NTf_2_, 2.5 equiv) in nitromethane to produce complex **1**. The ^1^H NMR spectrum of **1**, however, was broad at room temperature. Variable-temperature NMR (VT-NMR) revealed a set of sharp peaks at 358 K (Figure 1b, S9–S12). All ^1^H NMR signals assigned to **1** displayed the same diffusion coefficient in diffusion-ordered spectroscopy (DOSY), with a hydrodynamic radius of 15.5 Å, excluding the possibility that a mixture of different assemblies with different compositions was formed (Figure S16). The electrospray ionization mass spectrum (ESI-MS) of **1** exhibited a set of peaks corresponding to a composition Li_5_ **L**_2_ (Figures S14 and S15). All of these data are consistent with the formation of a Li_5_ **L**_2_ sandwich structure for **1**.

Furthermore, titrating a CD_3_CN solution of LiNTf_2_ into a CDCl_3_ solution of **L** resulted in precipitation, consistent with complex formation. Addition of CD_3_CN, producing a mixed solvent with the composition CDCl_3_/CD_3_CN, v/v = 9:4, dissolved the precipitate, which was also confirmed to be **1**. (Figure S17). ^7^Li NMR at 298 K (Figure S13) provided a set of overlapping peaks, which we attributed to the presence of multiple nearly isostructural configurations. Deconvolution of this peak cluster revealed three major signals with integral ratios of 2:2:1. We infer that this 2:2:1 ratio arises from the asymmetry of the pyrrole core,^51^ where the relative positions of the two pyrrole nitrogen atoms are inferred to give rise to slightly different local environments for the Li^+^ cations. Since these diastereomeric conformations have similar energies, the overall complex behaves as a dynamic system, giving rise to broad ^1^H NMR and ^7^Li NMR spectra (Table S4).

Despite more than 100 crystallization attempts under varied conditions, including different solvents, temperatures, concentrations, and counterions, we were unable to obtain single crystals of complex **1** suitable for X-ray diffraction analysis. As a result, the structural assignment of complex **1** is based on a combination of experimental techniques, including NMR spectroscopy and mass spectrometry, together with computational modeling. To identify the most favorable geometry, four MM3 ^54^ models of **1** were constructed based on the different coordination possibilities for Li–N (Figure S46). We then carried out energy minimizations at the GFN2-xTB level for different possible configurations of **1**.^55–57^ The configuration shown in Figure S47 (Table S2, **1a**) was found to have the lowest energy, suggesting that sandwich **1** may adopt this configuration. We then conducted density functional theory (DFT) minimization at the r^2^SCAN-3c level^58^ on this structure (Figure 1c), obtaining average Li–N distances ranging from 2.1–2.5 Å (Figure S51).

We also synthesized pentakis­(bidentate) azopyridine-based ligand **L′** (Scheme S2) in order to test its ability to bind lithium. The assembly of **L′** with LiNTf_2_ in nitromethane, however, did not yield an isolable discrete structure. The ^1^H NMR peaks of the product were very broad both at room temperature and at 363 K (Figure S18). The DOSY spectrum indicated a hydrodynamic radius of 24.0 Å, inconsistent with the presence of a sandwich structure (Figure S19). ESI-MS data also did not show formation of a Li_5_ **L′**_2_ product; instead, many fragments were observed. We thus infer that the tridentate azobipyridine groups of **L**, with each arm containing three nitrogen atoms to bind lithium are important for stabilizing the resulting sandwich structure.

We performed UV–vis titrations to calculate the binding affinity of Li^+^ for ligand **L**. A mixture of CHCl_3_ and CH_3_CN (v/v = 9:4) was used to prevent **1** from precipitating. Given that this system has a binding stereometry of 2:5, it is challenging to fit the titration data using common 1:1 or 1:2 binding curves. Therefore, we fit the titration data using a Hill plot,^59,60^ which allowed us to obtain the apparent association constant *K*_a_ = 2.88 × 10^10^ (mol dm^–3^)^−6^ (Figures S32 and S33). The Hill coefficient was calculated to be 2.01 (correlation coefficient: *R* ^2^ = 0.99), indicating positive cooperativity. This positive cooperativity indicates that the binding of the first Li^+^ to the ligands promotes the preorganization of two ligands to form the sandwich structure, making the binding of subsequent Li^+^ ions progressively easier until the final complex forms.^61^

Ligand *trans*-**L**, stable in the dark, was tested for its photoswitching behavior. Irradiation at 350 nm isomerized its *trans* azo groups to their *cis* configurations, as confirmed by a series of new peaks in the ^1^H NMR spectrum. Partially isomerized samples of **L** exhibited complex ^1^H NMR spectra due to the presence of many isomers (Figure S25 and S27). These complex NMR spectra render quantitative analysis of the O state (PSS) challenging. UV–vis spectra indicated that the PSS was reached after 90 s at 350 nm (Figure S26), and the process was reversed under irradiation at 575 nm.

We then investigated the photoswitching behavior of **1**, where irradiation with 350 nm light again caused the *trans*-azobipyridine moieties of *trans*-**1** to undergo photoisomerization to *cis*. This photochemical transformation was monitored by using both ^1^H NMR and ^7^Li NMR. As shown in Figure S29, a different ^1^H spectrum was observed after irradiation. Although the broadness of the peaks complicated analysis, DOSY analysis gave a hydrodynamic radius of 24.9 Å, which is larger than for **1** (Figure S31). The ^7^Li NMR spectrum (Figure S30) also revealed a sharp peak near 4 ppm after irradiation, suggesting the presence of partially dissociated Li^+^, while broad peaks at around 3.5 ppm correspond to Li^+^ ions that are still bound to the ligand. We infer that upon irradiation, photoisomerization of the azobipyridine groups on the ligand occurs from the *trans* form to the *cis* form, resulting in weaker lithium binding by the ligand arms in the *cis* form, as illustrated in Figure 2. UV–vis spectra show that the PSS is reached after 120 s (Figure S28).

**Figure 2:**
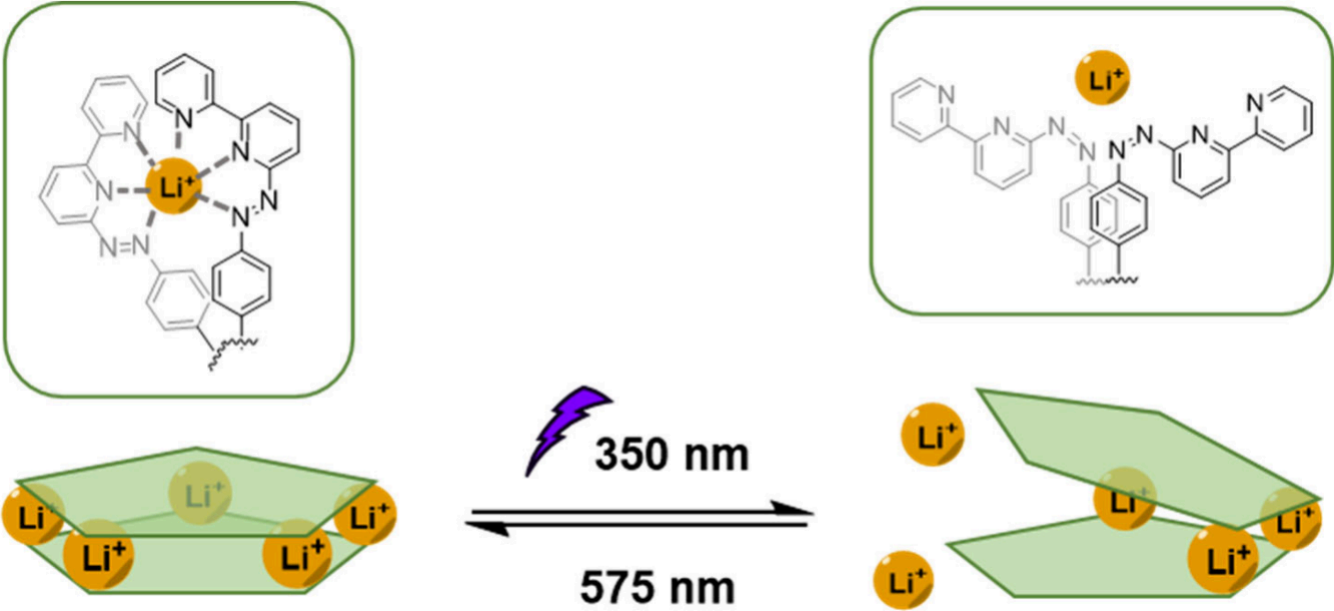
Cartoon illustration of the photoswitching behavior of the Li_5_ **L**_2_ sandwich **1**.

We then investigated the formation of complexes with other alkali metal ions, including Na^+^ and K^+^. Ligand **L** (1 equiv) and sodium or potassium­(I) bis­(trifluoromethanesulfon)­imide (triflimide or ^–^NTf_2_, 2.5 equiv) were dissolved in nitromethane, and the resulting suspension was stirred at 343 K. After heating for 3 days, substantial precipitate was observed, suggesting that neither Na^+^ nor K^+^ formed a nitromethane-soluble complex with **L** (Figure S38). Analogous experiments in CDCl_3_ and CD_3_CN mixed solution (Figure S39) likewise did not indicate interaction with ligand **L**. UV–vis titrations were performed to determine the *K*_a_ value using a Hill plot. As shown in Figure S34–S37, the *K*_a_ values of Na^+^ and K^+^ are 10^5^ times lower than that of Li^+^. In addition, the selective binding affinity was evaluated using the tetrafluoroborate (BF_4_^–^) conterion. LiBF_4_ formed a nitromethane-soluble complex with ligand **L** (Scheme S5, Figures S20–S22), no soluble products were observed with **L** and NaBF_4_ or KBF_4_ in CH_3_NO_2_. Hence, these results are consistent with weaker binding affinity of Na^+^ and K^+^ toward tridentate ligand **L** compared to Li^+^, such that the strength of metal binding is unable to overcome the noncovalent interactions holding molecules of **L** together in the solid state.

We envisaged that the observed selectivity of the system for Li^+^ might enable the extraction of Li^+^ from mixtures of other alkali metal ions. We first investigated extraction using Li^+^ alone. As shown in Figure S41, ligand **L** was dissolved in CDCl_3_, followed by the addition of Li­(NTf_2_)_2_ in CD_3_CN (CDCl_3_/CD_3_CN, v/v = 20:1), resulting in the formation of **1** as a precipitate. After the supernatant was decanted, this precipitate was redissolved in CD_3_NO_2_. Upon irradiation at 350 nm, we did not observe precipitation. However, after the subsequent addition of water, a precipitate was observed, consistent with the dissociation of Li^+^ and the precipitation of ligand **L** from nitromethane. The addition of water without initial irradiation did not generate precipitates. Thus, the illumination and addition of water led to the decomposition of Li_5_ **L**_2_ complex **1** and the release of free Li^+^.

Our results enabled the development of a five-step process for the extraction of lithium from the other alkali metal ions (Figure 3). First, ligand **L** was dissolved in CHCl_3_. A mixture of LiNTf_2_, KNTf_2_ and NaNTf_2_ in CH_3_CN solution (solution **a**) was added to the **L** solution, where the lithium bound selectively to **L** to form complex **1**, which precipitated. The unreacted K^+^ and Na^+^ ions remained in the supernatant and were separated as solution **b**. The precipitate of **1** was then dissolved in CH_3_NO_2_. Next, water was added, and the mixture was illuminated at 350 nm for 30 min. This treatment resulted in the decomposition of **1**, with the precipitation of ligand **L** and extraction of Li^+^ ions into the water phase (solution **c**). Note that both water and illumination were necessary for Li^+^ extraction. After Li^+^ extraction, the *trans* form of ligand **L** was regenerated by irradiation at 575 nm for 60 min, enabling it to be recycled.

**Figure 3:**
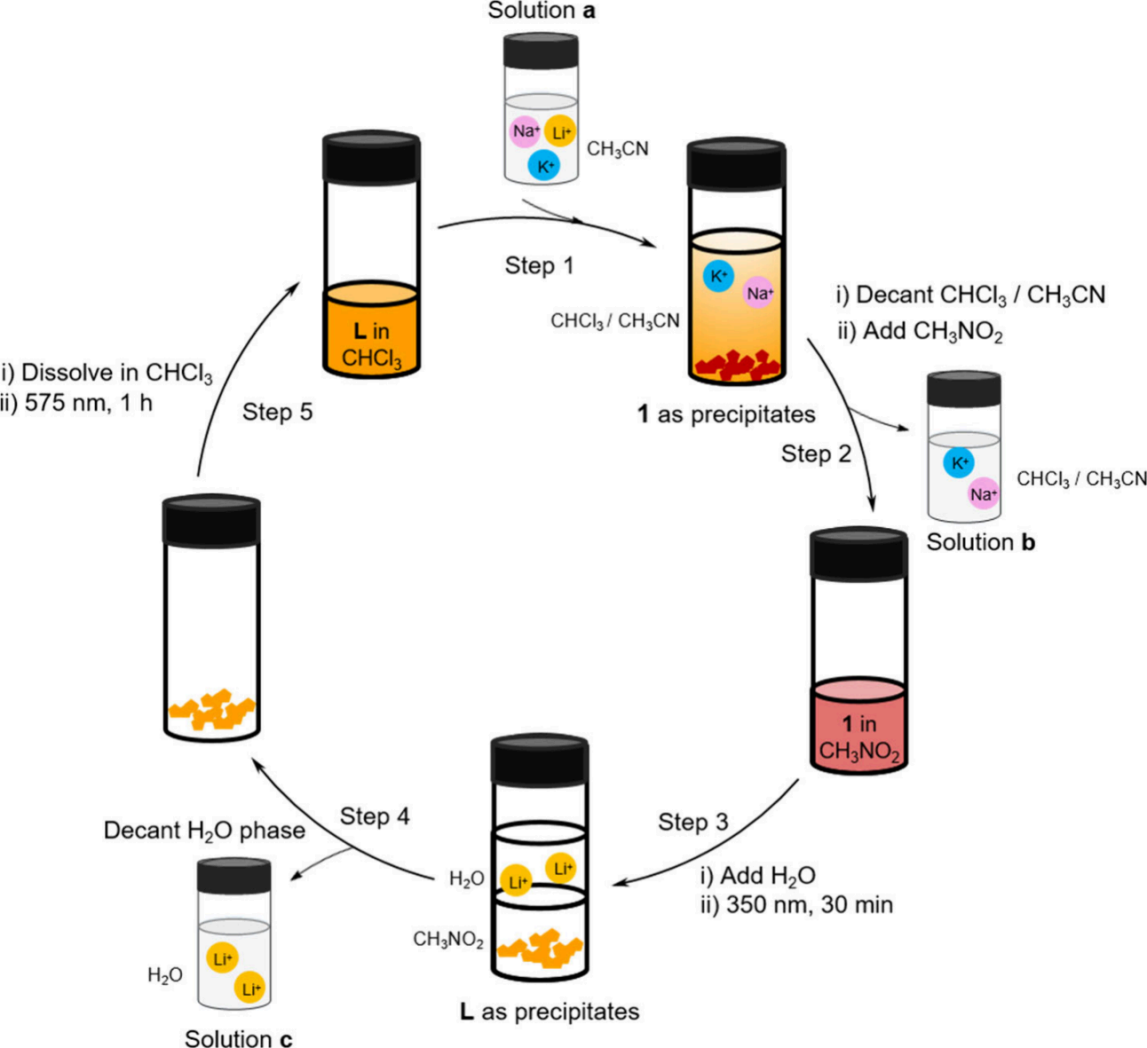
Schematic representation of lithium extraction. Step 1: Ligand **L** was dissolved in CHCl_3_, and an equimolar mixture of LiNTf_2_, KNTf_2_ and NaNTf_2_ in CH_3_CN (solution **a**) is added to the **L** solution, resulting in the coordination of the lithium ion to **L** to form complex **1** as a precipitate. Step 2: The unreacted K^+^ and Na^+^ ions remained in the supernatant and were separated as solution **b**. The precipitate of **1** was then dissolved in CH_3_NO_2_. Step 3: Water was added, and the mixture was illuminated at 350 nm for 15 min. This process resulted in the decomplexation of **1**, with **L** precipitating and Li^+^ extracted into the water phase (solution **c**). Step 4: The water phase containing Li^+^ was separated. Step 5: Ligand **L** was recycled after illumination at 575 nm for 60 min.

This extraction cycle was monitored by inductively coupled plasma optical emission spectroscopy (ICP-OES). In the original solution **a**, the concentration of each metal cation was normalized to 100%. After forming sandwich structure **1**, solution **b** contained 91% (±6.6%) of the original Na^+^ and 87% (±3.5%) of K^+^, while 24% (±1.6%) of Li^+^ remained in this solution. After irradiation at 350 nm and extraction with water, solution **c** contained 76% (±1.6%) of the original Li^+^ (Figure S42 and Table S1). These results demonstrate efficient Li^+^ ion extraction from an alkali metal mixture. A stability test monitored by ^1^H NMR (Figure S43) showed that approximately 80% of ligand **L** was recovered after the first cycle. However, only 33.3% was recovered after five cycles, likely due to a partial loss of the ligand and complex during repeated decantation steps.

This work thus demonstrates a lithium extraction method that utilizes the reversible formation of photoresponsive metal–organic architecture **1**, where Li^+^ ions are selectively and efficiently extracted from a mixture of alkali metal ions by forming Li_5_ **L**_2_ complex **1**. This technique offers a potentially useful new path to lithium extraction, paving the way for cleaner, more environmentally friendly modes of resource extraction and recycling. Partial photoisomerization of multivalent ligand **L** is sufficient to disrupt the complex and release lithium, demonstrating the effectiveness of responsive assemblies. Subsequent designs that incorporate more efficient photoresponsive units, potentially tethered to a solid surface to avoid their loss, could enhance the practical applicability of this concept.^62,63^

## Supplementary Material



## Data Availability

The Python code for performing the computational study and input and output structures are available at https://github.com/PaulaTeeuwen/Photo-Driven-Li-Extraction-Cages.

## Notes

Experimental procedures; NMR characterizations; mass spectrometry data; Any additional data related to this paper may be requested from the authors (PDF)
