# Supplementary material for: Light-Driven Lithium Extraction from Mixtures of Alkali Cations Using an Azobipyridine Ligand
Source: J Am Chem Soc. 2025 Jun 9;147(24):20205–11. doi: 10.1021/jacs.5c05885 (PMC12186520; doi:10.1021/jacs.5c05885)
Supplement: Supplementary file 1 [file ja5c05885_si_001.pdf]

Supplementary Materials for

Light-driven Lithium Extraction from Mixtures of Alkali Cations using an Azobipyridine Ligand

Yuyin Du[1]†, Amit Ghosh[1]†, Paula C. P. Teeuwen[1], David J. Wales[1]*, Jonathan R. Nitschke[1]*

[1]Yusuf Hamied Department of Chemistry, University of Cambridge; Cambridge, CB2 1EW, United Kingdom.

†These authors contributed equally to this work

*Corresponding author: dw34@cam.ac.uk (D.J.W.), jrn34@cam.ac.uk (J.R.N.)

Table of Contents

1. General Information **3**

2. Synthesis and Characterisation **4**

2.1 Synthesis of tridentate azobipyridine-containing ligand **L** 4

2.2 Synthesis of bidentate azopyridine-containing ligand **L’** 8

2.3 Construction and characterization of sandwich complex **1** 11

2.4 Self-assembly of **L’** with $LiNTf_2$ 19

2.5 Construction and characterization of the complex between **L** and $LiBF_4$ 22

2.6 Self-assembly of iminopyridine-based analogs 25

3. Investigation of photoswitching properties **26**

3.1 Photoswitching properties for ligand **L** 26

3.2 Photoswitching properties for complex **1** 28

4. Binding affinities of Li^+/K^+/Na^+ towards ligand L **31**

5. Selectivity of alkali metal ions **35**

6. Lithium Extraction cycle **37**

6.1 General procedures 37

6.2 Inductively Coupled Plasma Spectrometry (ICP) Analysis 38

6.3 Stability over five rounds recycling. 41

7. Geometry optimization and energy evaluations of sandwich complex 1 .... **42**

7.1 Enumeration of pentagonal sandwich diastereomers 42

7.2 Investigation of the Li-coordination environments 47

7.3 Investigation of the relative pyrrole-nitrogen positions 50

7.4 Investigation of relative orientations of the phenyl rings 52

8. References **53**

1. General Information

Materials and methods

Unless otherwise stated, all chemicals were obtained from commercial sources and used as received.

Nuclear Magnetic Resonance (NMR)

NMR experiments employed Bruker AVANCE III and NEO (400 and 500 MHz) spectrometers. Chemical shifts for ^{1}H and ^{13}C NMR are reported in ppm with residual solvent as reference: nitromethane (4.33 ppm for ^{1}H), chloroform (7.26 ppm for ^{1}H, 77.22 ppm for ^{13}C). Abbreviations for signal multiplicity of ^{1}H NMR spectra are shown as following: s: singlet, d: doublet, t: triplet, dd: doublet of doublets; dt: doublet of triplets; m: multiplet, br: broad.

Mass spectrometry (MS)

High-resolution electrospray ionization (HR-ESI) mass spectra were obtained with Waters Synapt G2-Si instrument (cone voltage 30 eV; desolvation temperature 353 K; ionization temperature 373 K) infused from a Harvard Syringe Pump at a rate of 10 μL per minute.

Photoirradiation

Irradiation at 350 nm and 575 nm was performed in situ by placing the NMR tubes inside a Rayonet photochemical chamber reactor (40 cm deep, 25 cm diameter, 16 × 14 W light sources, operating temperature 32 °C).

2. Synthesis and Characterisation

2.1 Synthesis of tridentate azobipyridine-containing ligand **L**

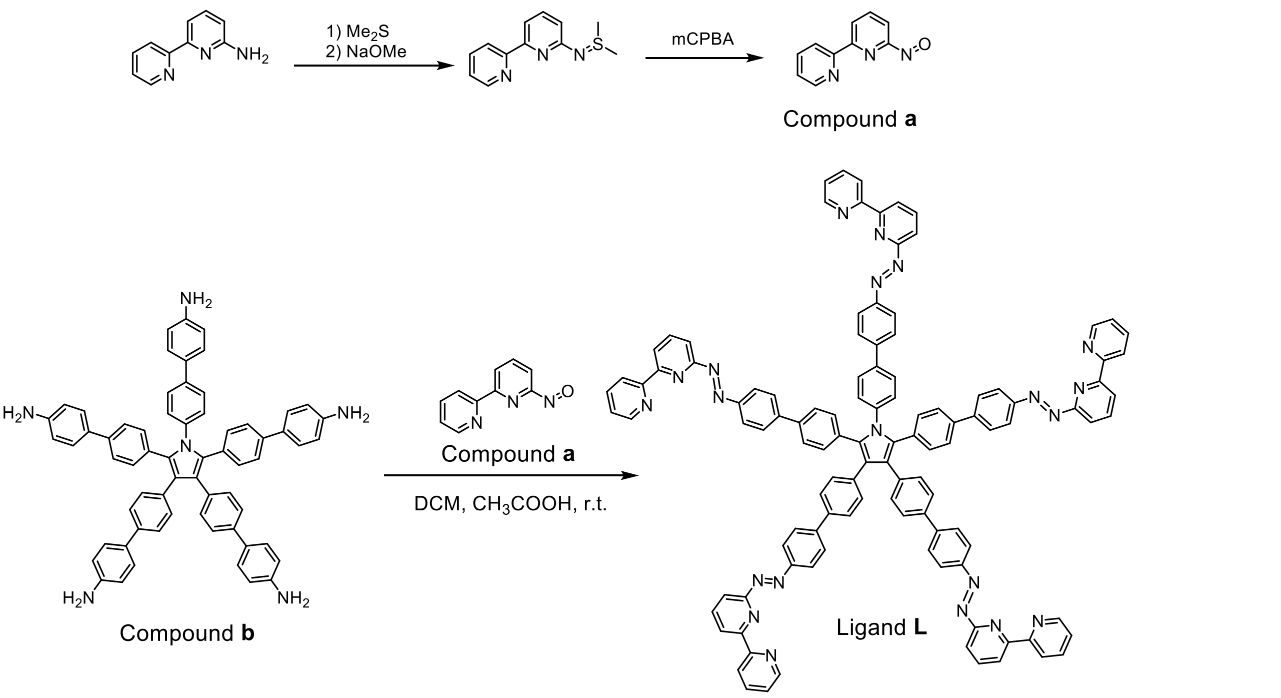

Scheme S1. Synthesis of tridentate ligand **L**.

Compounds **a** and **b** were synthesized according to the literature.[51,64]

Compound **b** (130.0 mg, 1 equiv.) and 6-nitroso-2,2'-bipyridine (399.9 mg, 15 equiv.) were added to a round-bottom flask, after addition of a mixed solution of CH_2Cl_2/Glacial acetic acid (15 mL, v/v = 4:1). The reaction mixture was stirred at room temperature for 72 hours. The solvent was removed *in vacuo*, and the crude product was dissolved in CH_2Cl_2 (50 mL) and washed with saturated sodium carbonate solution (50 mL). The organic phase was collected, dried over Na_2SO_4, filtered, and concentrated *in vacuo*. The crude product was further washed with methanol (3 x 30 mL), and dried to afford an orange solid (90.98 mg, 84.4% yield).

^{1}H NMR (500 MHz, 298K, $CDCl_3$) δ 8.71 (d, 5H), 8.65 (dd, 5H), 8.56 (dd, 5H), 8.14 (dt, 10H), 8.02 (t, 5H), 7.82 (m, 20H), 7.58 (m, 10H), 7.35 (m, 5H), 7.18 (m, 10H)

^{13}C NMR (126 MHz, 298K, $CDCl_3$) δ 163.19, 163.14, 163.08, 156.21, 156.18, 156.15, 155.44, 155.41, 155.38, 151.63, 151.47, 151.32, 149.19, 149.18, 149.17, 144.59, 144.07,

143.39, 139.18, 139.16, 139.15, 138.51, 138.35, 137.89, 137.88, 137.01, 137.00, 136.89, 135.13, 131.89, 131.83, 131.80, 131.66, 129.63, 127.68, 127.51, 127.42, 127.33, 126.66, 126.60, 124.28, 124.25, 124.12, 124.10, 124.08, 123.18, 122.56, 122.47, 121.78, 121.76, 113.08, 113.02, 113.00.

ESI-MS (CH_3CN) *m/z*: $[M+H]^+$ calc. for $C_{114}H_{75}N_{21}$, 1739.968, found 1739.656.

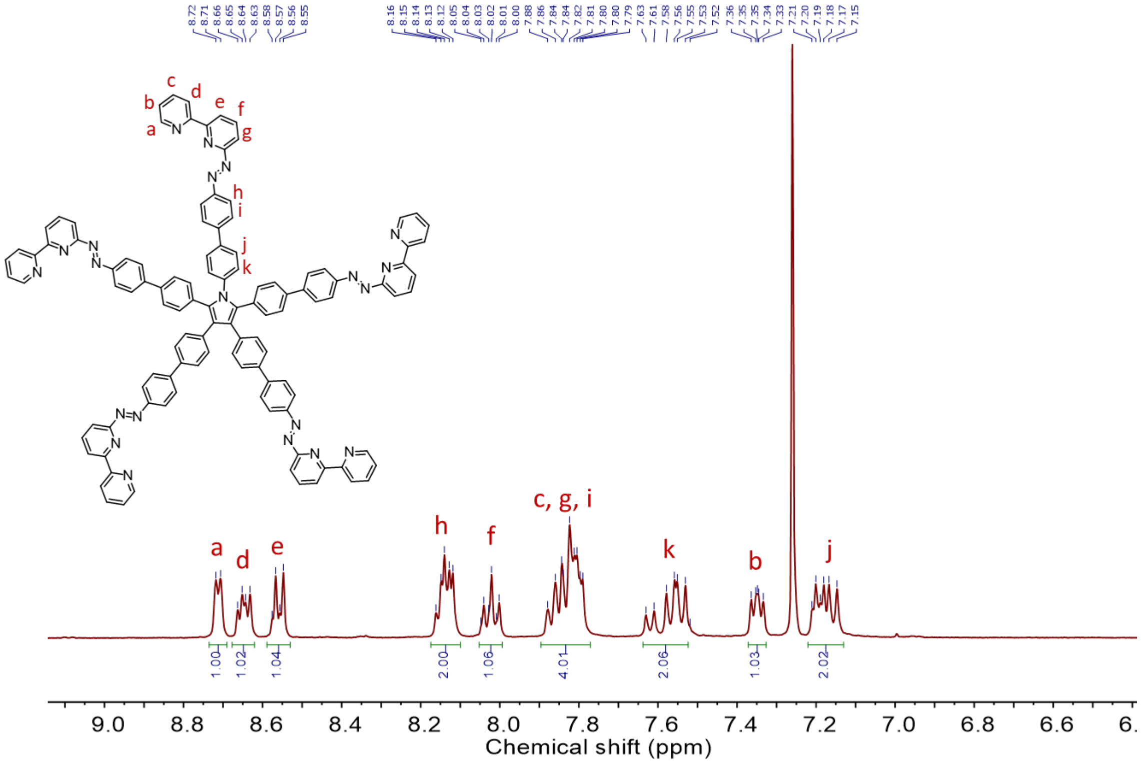

Figure S1. ^1H NMR spectrum (500 MHz, 298K, $CDCl_3$) of ligand **L**.

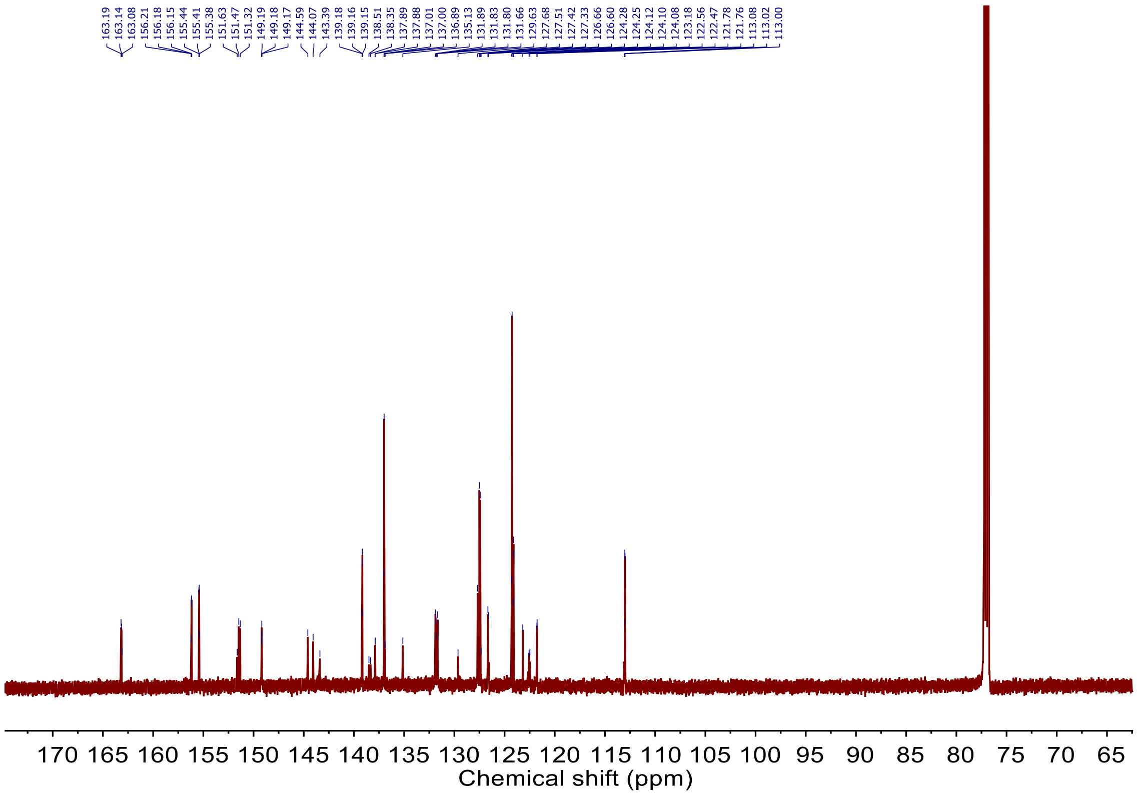

Figure S2. ^{13}C NMR spectrum (126 MHz, 298K, $CDCl_3$) of ligand **L**.

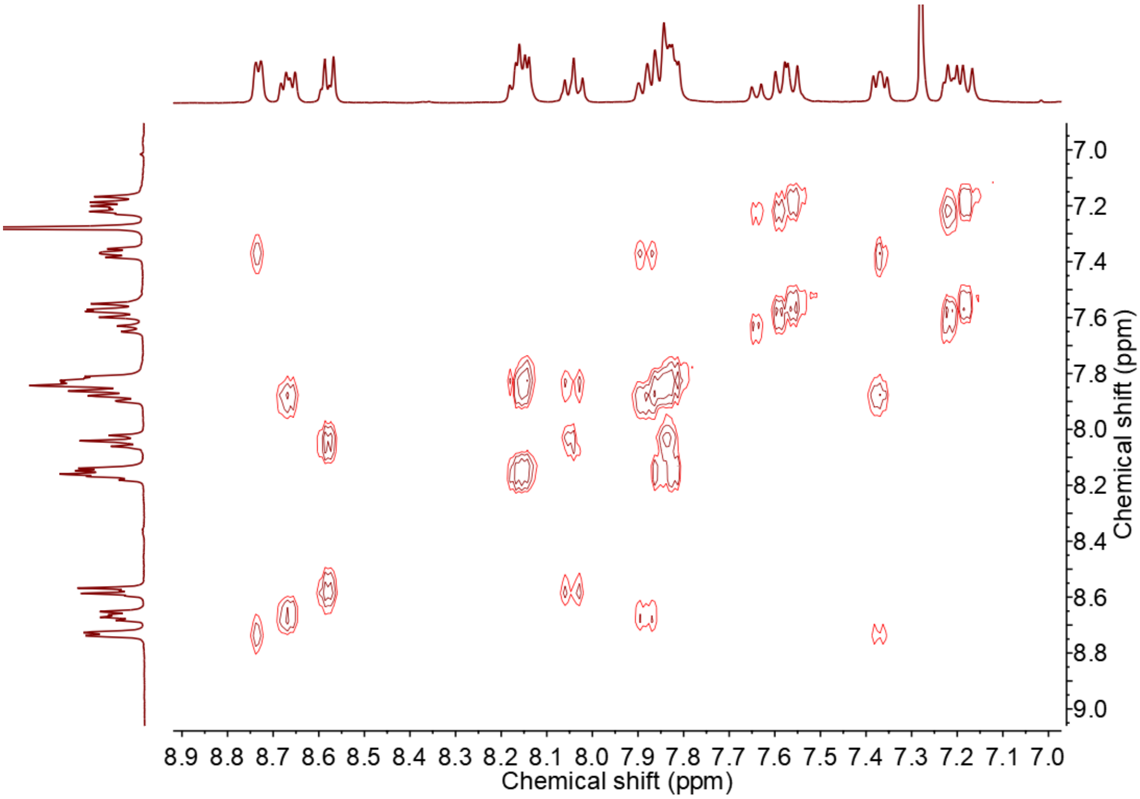

Figure S3. Partial ^{1}H-^{1}H COSY NMR spectrum (500 MHz, 298K, $CDCl_3$) of ligand **L**.

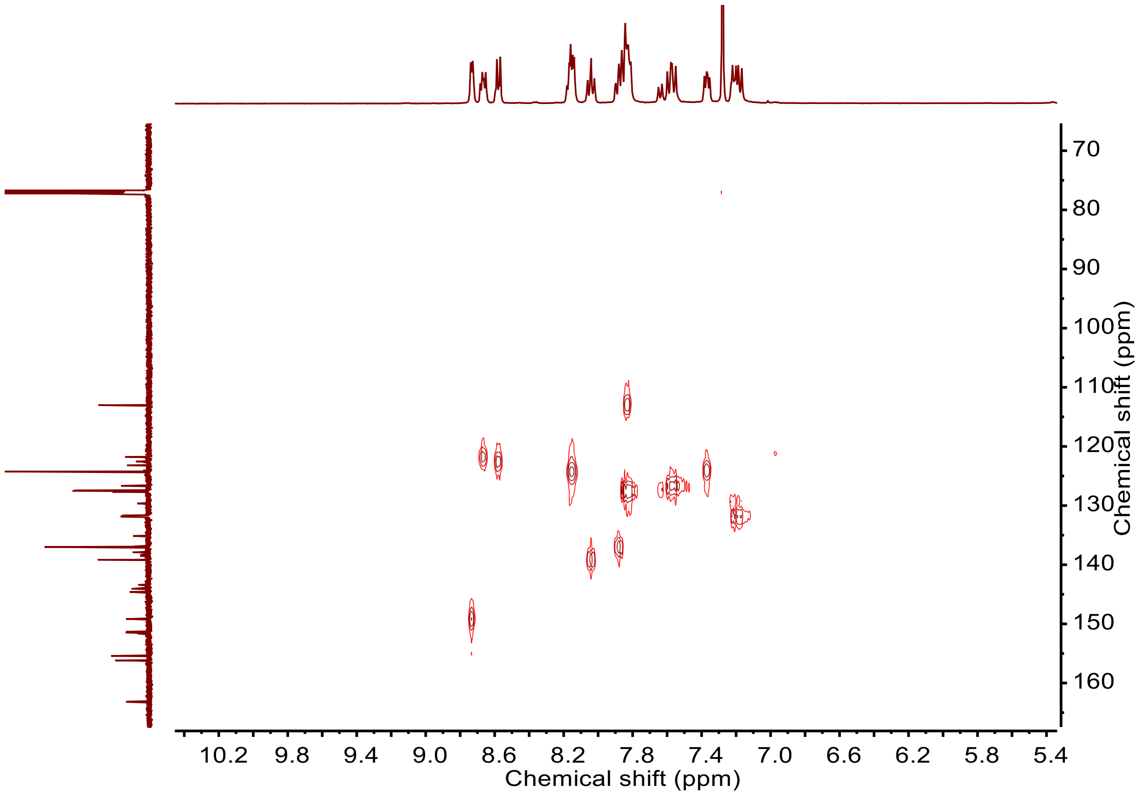

Figure S4. Partial ^{1}H-^{13}C HSQC NMR spectrum (500 MHz, 298K, $CDCl_3$) of ligand **L**.

2.2 Synthesis of bidentate azopyridine-containing ligand **L’**

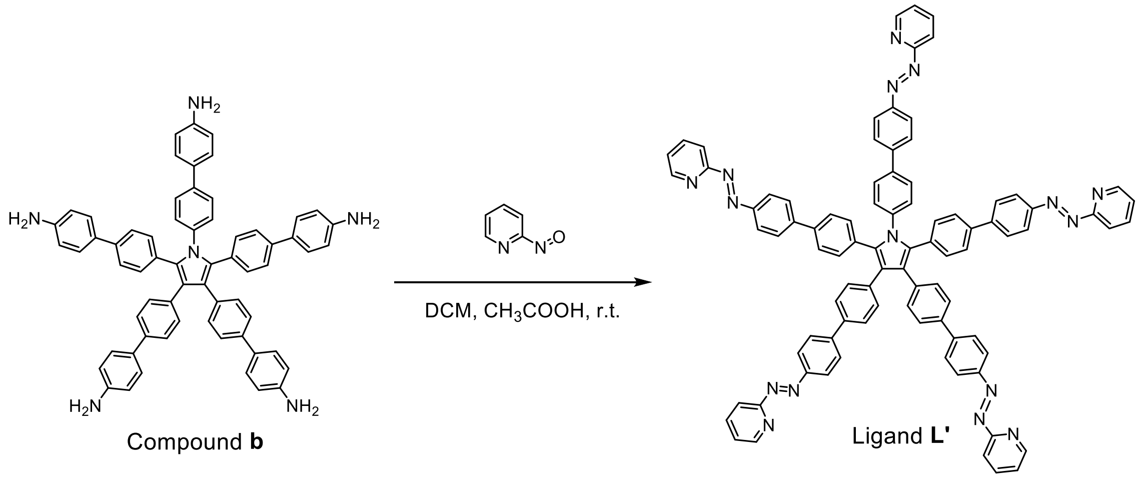

Scheme S2. Synthesis of bidentate ligand **L’**.

Compound **b** (86.4 mg, 1 equiv.) and 2-nitrosopyridine (155.1 mg, 15 equiv) were added to a round-bottom flask containing a mixed solution of CH_2Cl_2/Glacial acetic acid (10 mL, v/v = 4:1). The reaction mixture was stirred at room temperature for 72 hours. The solvent was removed *in vacuo*, and the crude product was dissolved in CH_2Cl_2 (50 mL) and washed with saturated sodium carbonate solution (50 mL). The organic phase was collected, dried over Na_2SO_4, filtered, and concentrated *in vacuo*. The crude product was further washed by methanol (3 x 30 mL), and dried to produce an orange solid (103.6 mg, 80.3% yield).

^{1}H NMR (500 MHz, $CDCl_3$) δ 8.71 (d, 5H), 8.65 (dd, 5H), 8.56 (dd, 5H), 8.14 (dt, 10H), 8.02 (t, 5H), 7.82 (m, 20H), 7.58 (m, 10H), 7.35 (m, 5H), 7.18 (m, 10H)
^{13}C NMR (126 MHz, $CDCl_3$) δ 163.02, 162.97, 162.91, 151.69, 151.38, 149.61, 149.58, 149.57, 144.57, 144.04, 143.37, 138.51, 138.35, 138.33, 138.31, 137.88, 136.89, 135.10, 131.86, 131.80, 131.77, 131.63, 129.61, 127.70, 127.53, 127.49, 127.44, 127.29, 126.63, 126.56, 125.28, 125.19, 125.11, 124.21, 124.17, 123.15, 115.72, 115.60, 115.55.
ESI-MS (CH_3CN) *m*/*z*: $[M+H]^+$ calc. for $C_{89}H_{60}N_{16}$, 1353.539, found 1353.523.

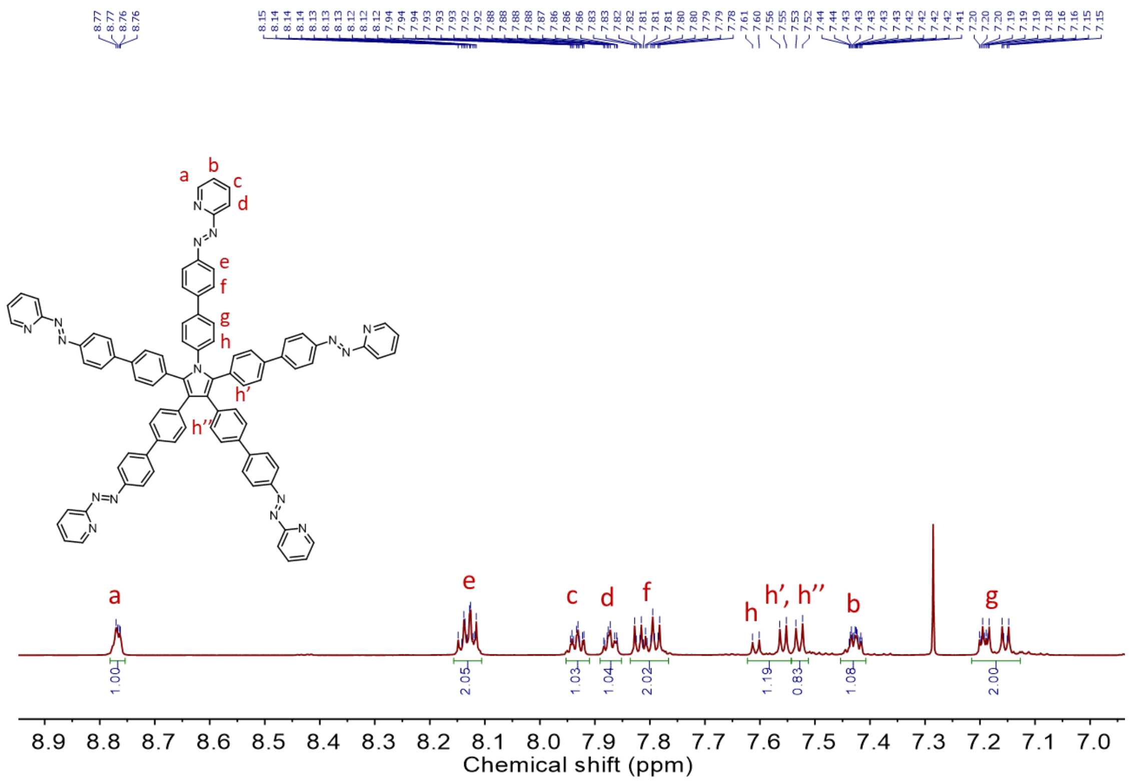

Figure S5. ^{1}H NMR spectrum (500 MHz, 298 K, $CDCl_3$) of ligand **L'**.

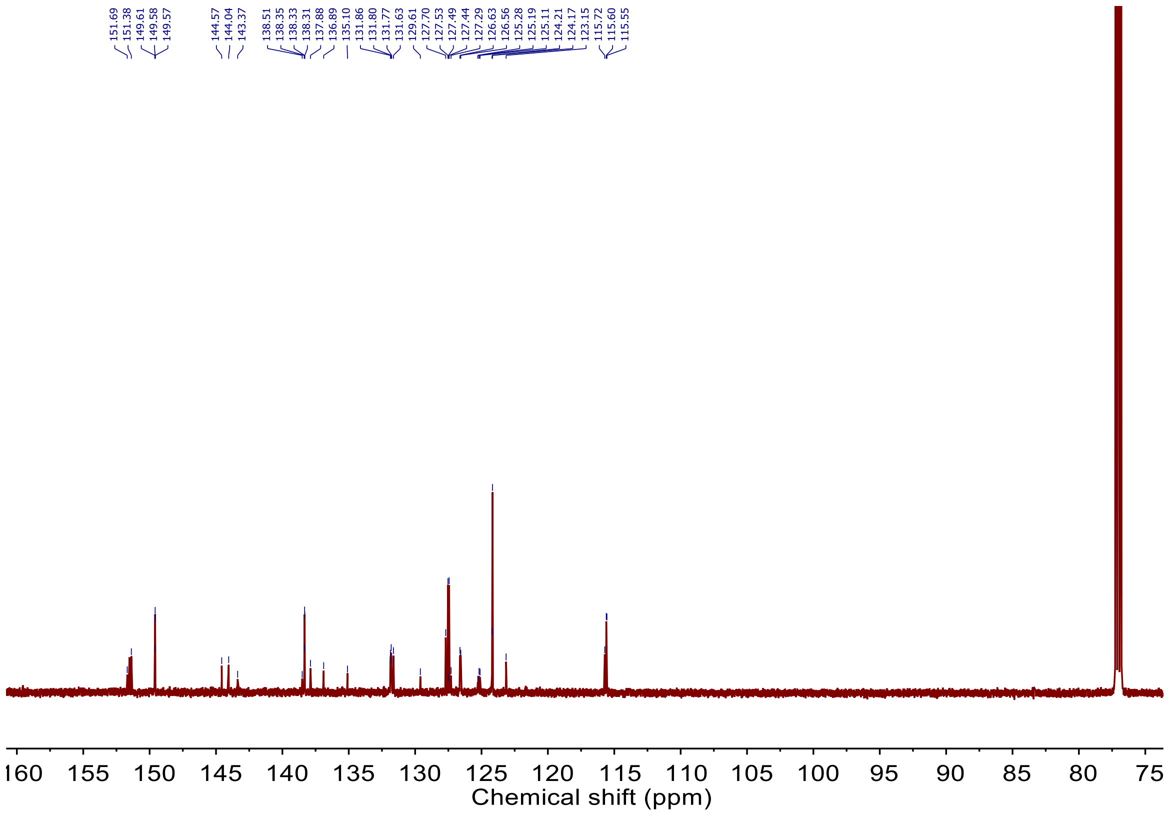

Figure S6. ^{13}C NMR spectrum (126 MHz, 298 K, $CDCl_3$) of ligand **L'**.

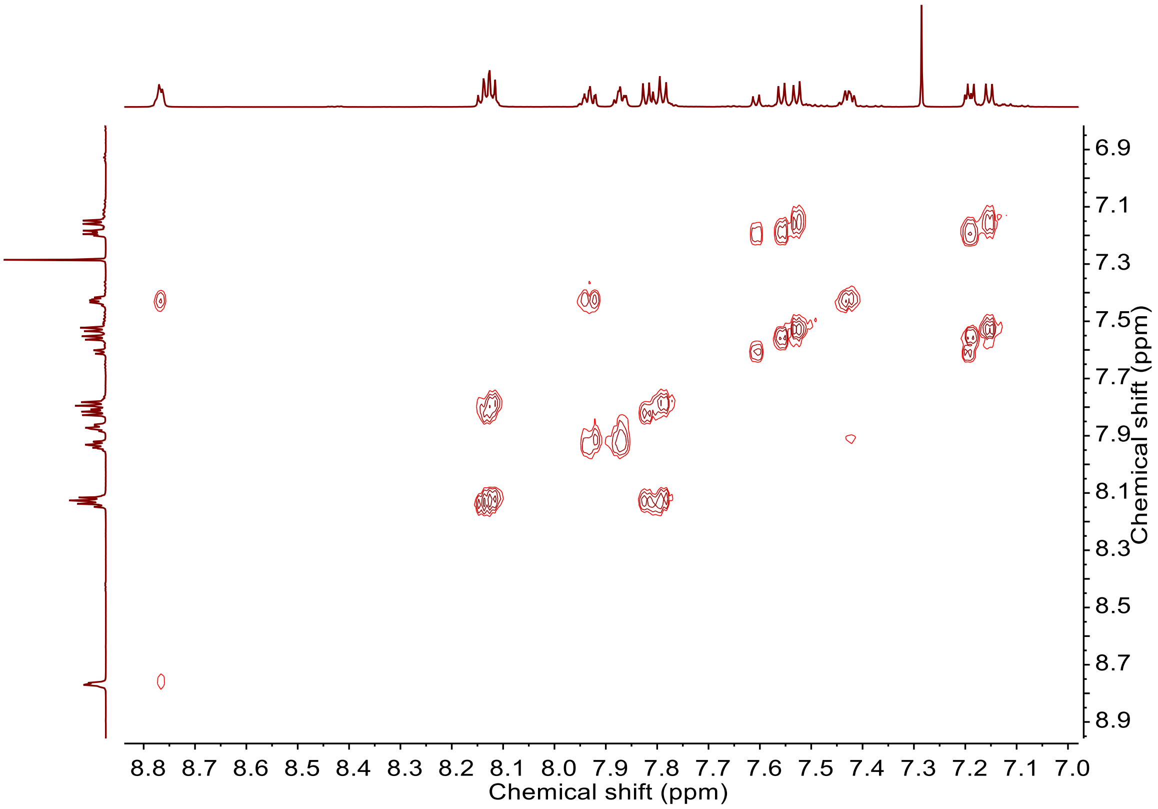

Figure S7. Partial ^{1}H-^{1}H COSY NMR spectrum (500 MHz, 298K, $CDCl_3$) of ligand **L'**.

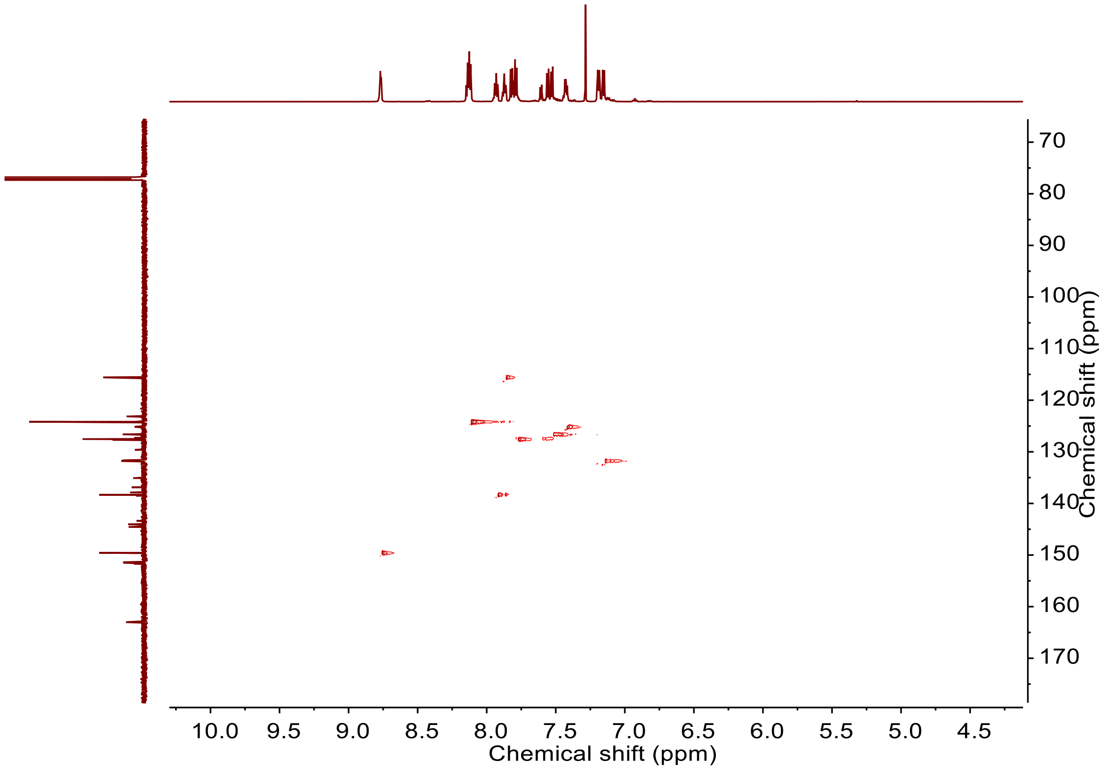

Figure S8. Partial ^{1}H-^{13}C HSQC NMR spectrum (500 MHz, 298K, $CDCl_3$) of ligand **L'**.

2.3 Construction and characterization of sandwich complex **1**

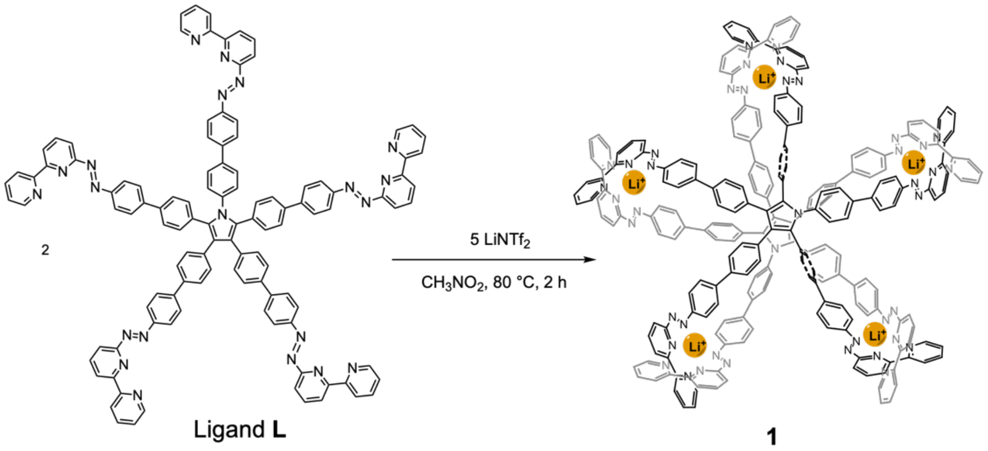

Scheme S3. Self-assembly of sandwich complex **1**.

Ligand **L** (1.0 mg, 0.58 µmol, 1 equiv.), $LiNTf_2$ (0.42 mg, 1.45 µmol, 2.5 equiv.) and CH_3NO_2 (0.4 ml) were added to a vial, and the mixture was sonicated for 1 min. The mixture was then heated at 80 °C for 2 hours. The solvent was removed by blowing with N_2 and then Et_2O (10 ml) was added. The resulting solid was collected by centrifugation, washed three times with additional Et_2O (10 ml) and then dried under dynamic vacuum for 16 h at 298 K to give sandwich **1**$(NTf_2)_5$ as a red solid (1.28 mg, 0.26 µmol, yield 90 %).

^{1}H NMR (400 MHz, CD_3NO_2) δ 8.63 (s, 10H), 8.43 – 8.47 (m, 20H), 8.29 – 8.35 (m, 20H), 8.10 (s, 30H), 7.83 – 7.88 (m, 20H), 7.63 – 7.73 (m, 20H), 7.54– 7.57 (m, 10H), 7.39 – 7.42 (m, 20H).

ESI-MS (CH_3NO_2) *m/z* = 702.47 $[Li_5\mathbf{L}_2]^{5+}$, 948.07 $[Li_5\mathbf{L}_2(NTf_2)]^{4+}$, 1357.73 $[Li_5\mathbf{L}_2(NTf_2)_2]^{3+}$.

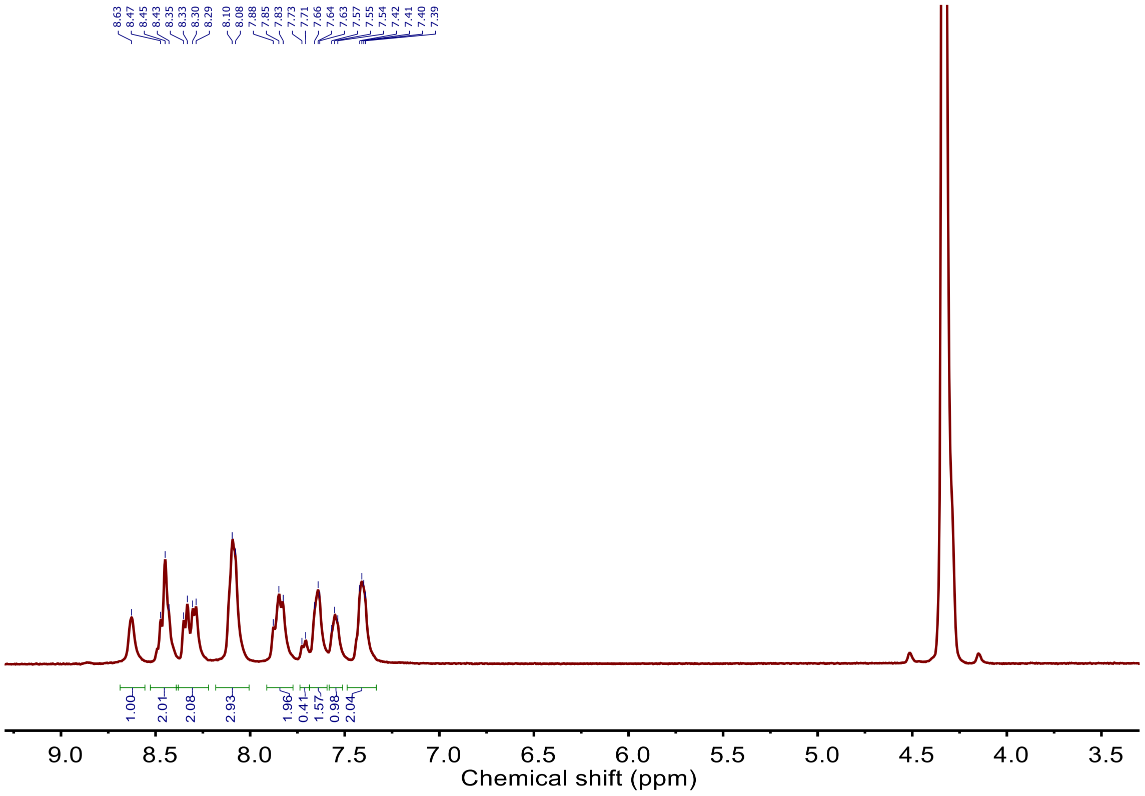

Figure S9. ^{1}H NMR spectrum (400 MHz, 363 K, CD_3NO_2) of complex **1**.

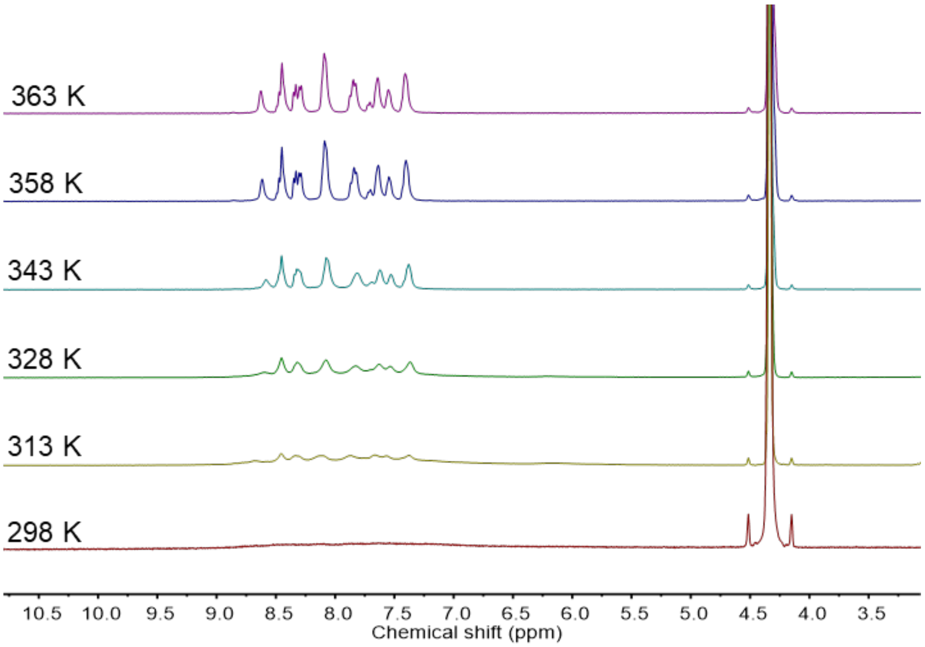

Figure S10. ^{1}H VT-NMR (400 MHz, CD_3NO_2) of complex **1** from 298 K to 363 K. Higher temperatures generated sharper peaks. Lowering the temperature to 253 K did not result in the observation of peaks.

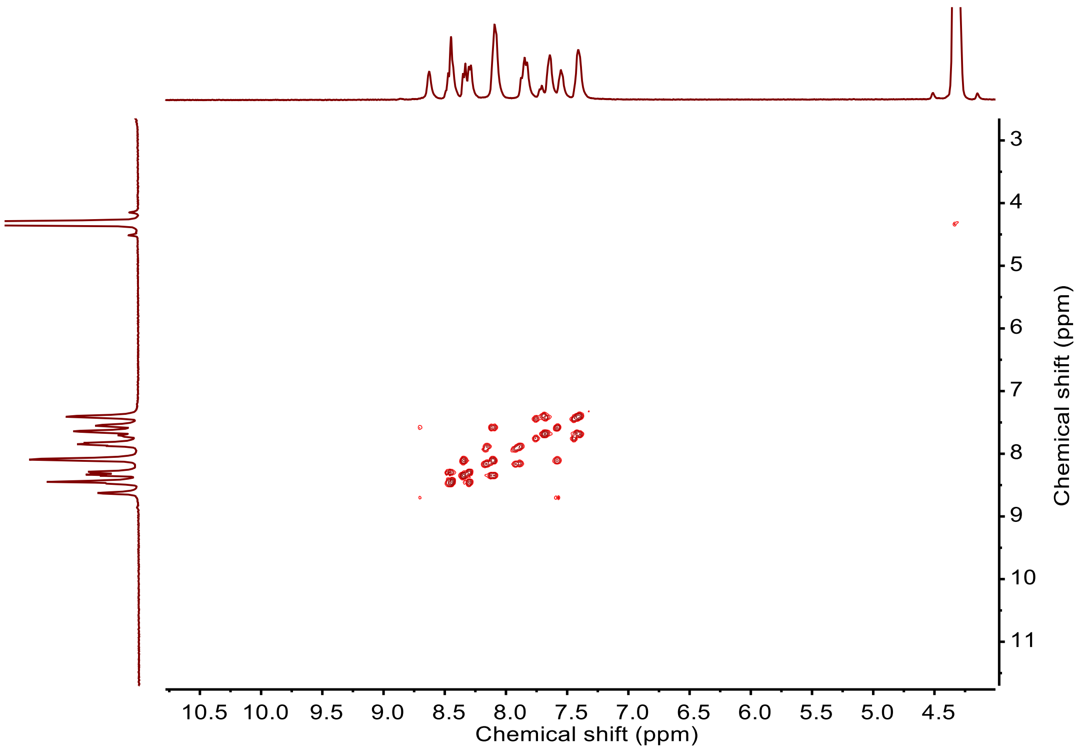

Figure S11. Partial ^{1}H-^{1}H COSY NMR spectrum (400 MHz, 363K, CD_3NO_2) of complex **1**.

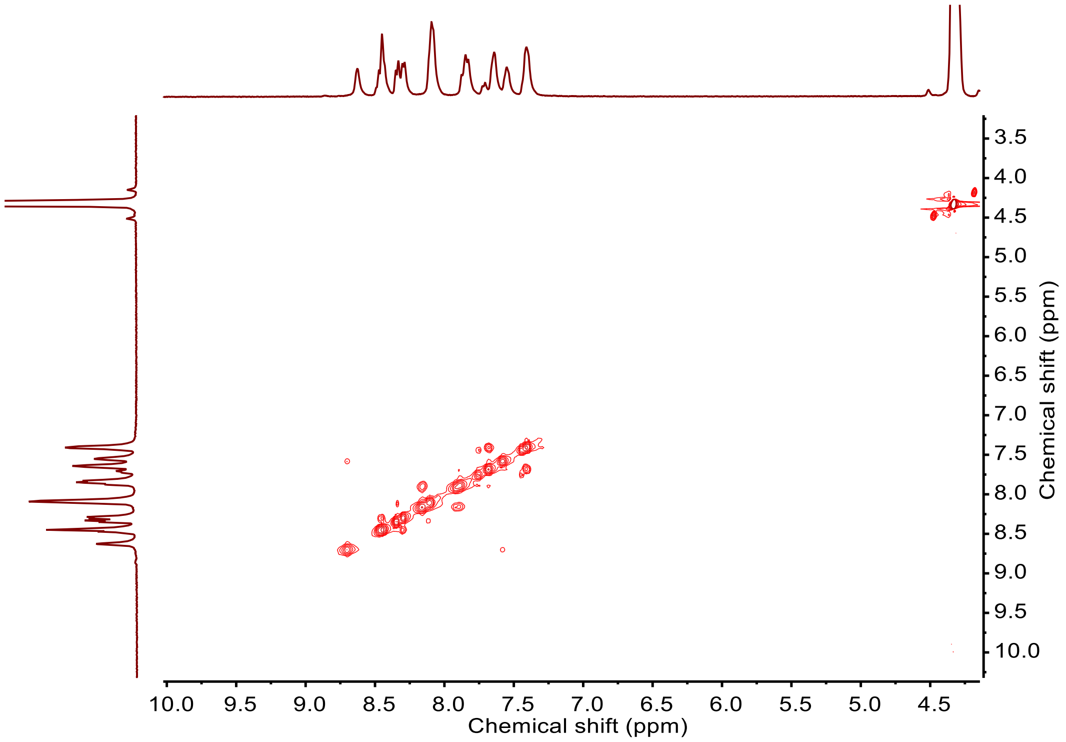

Figure S12. Partial ^{1}H-^{1}H NOESY NMR spectrum (400 MHz, 363K, CD_3NO_2) of complex **1**.

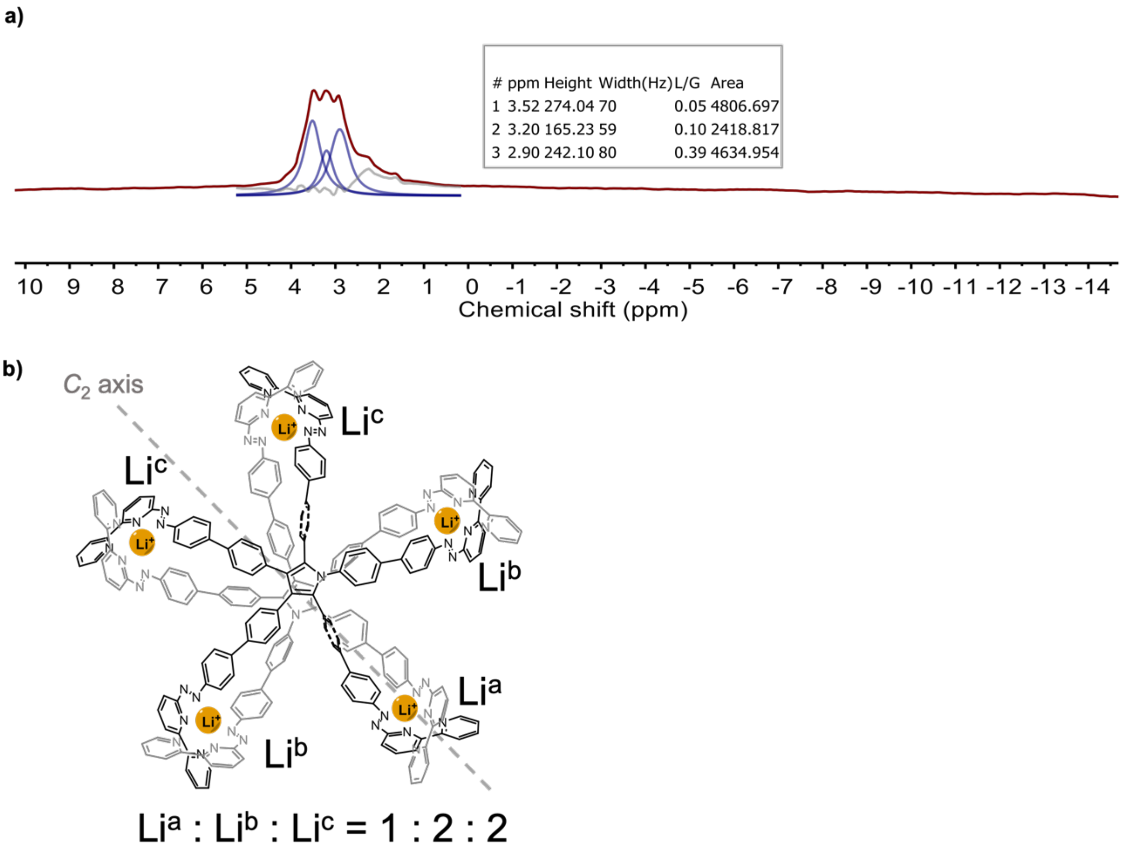

Figure S13. a) ^{7}Li NMR (194 MHz, 298K, CD_3NO_2) of complex Li_5**L**$_2$. Deconvolution was applied and peak areas for all three major peaks were analyzed. Peaks are broad and overlapped with each other due to the presence of many configurational isomers. The peak area ratio is roughly 2:1:2. **b)** Illustration of possible lithium environments according to the structure shown in **Figure 1c**. Due to the unsymmetrical properties of the pyrrole core, the overall structure has C_2 axis which creates 3 different Li^+ environments Li^a, Li^b and Li^c with 2:2:1 ratio.

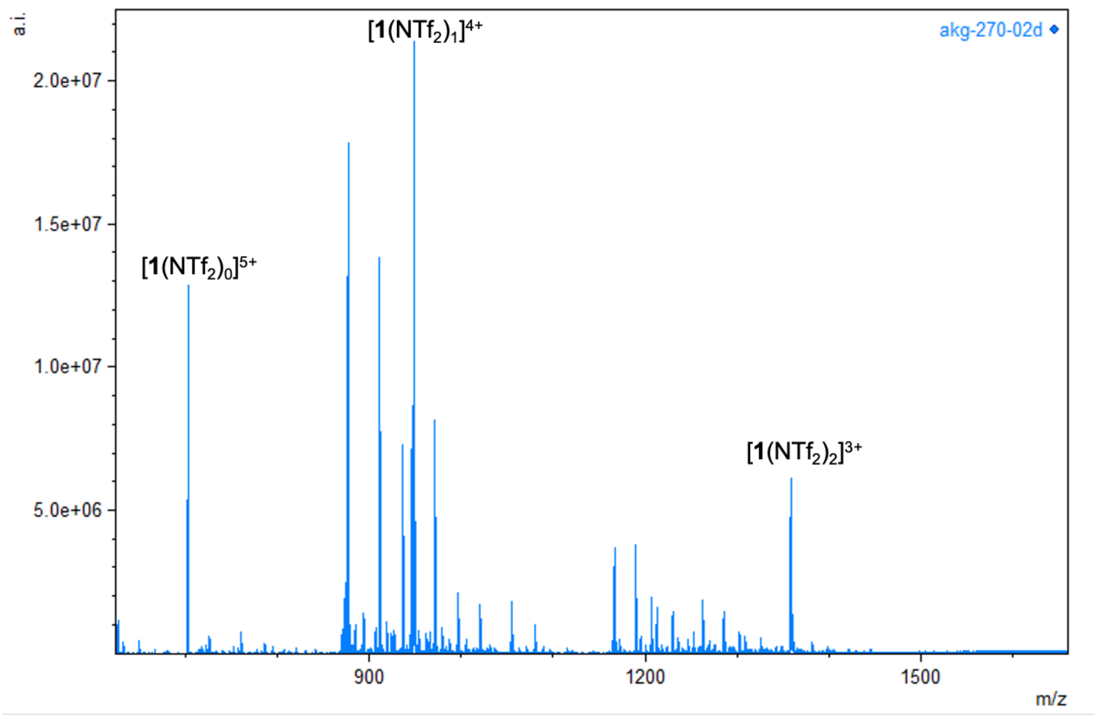

Figure S14: High-resolution ESI-mass spectrum of **1**·$[NTf_2]_5$. ESI-MS: Experimental results: *m/z* = 702.47 $[Li_5\mathbf{L}_2]^{5+}$, 948.07 $[Li_5\mathbf{L}_2(NTf_2)_1]^{4+}$, 1357.73 $[Li_5\mathbf{L}_2(NTf_2)_2]^{3+}$. Calculated results: *m/z* = 702.52 $[Li_5\mathbf{L}_2(NTf_2)_0]^{5+}$, 948.19 $[Li_5\mathbf{L}_2(NTf_2)_1]^{4+}$, 1357.64 $[Li_5\mathbf{L}_2(NTf_2)_2]^{3+}$.

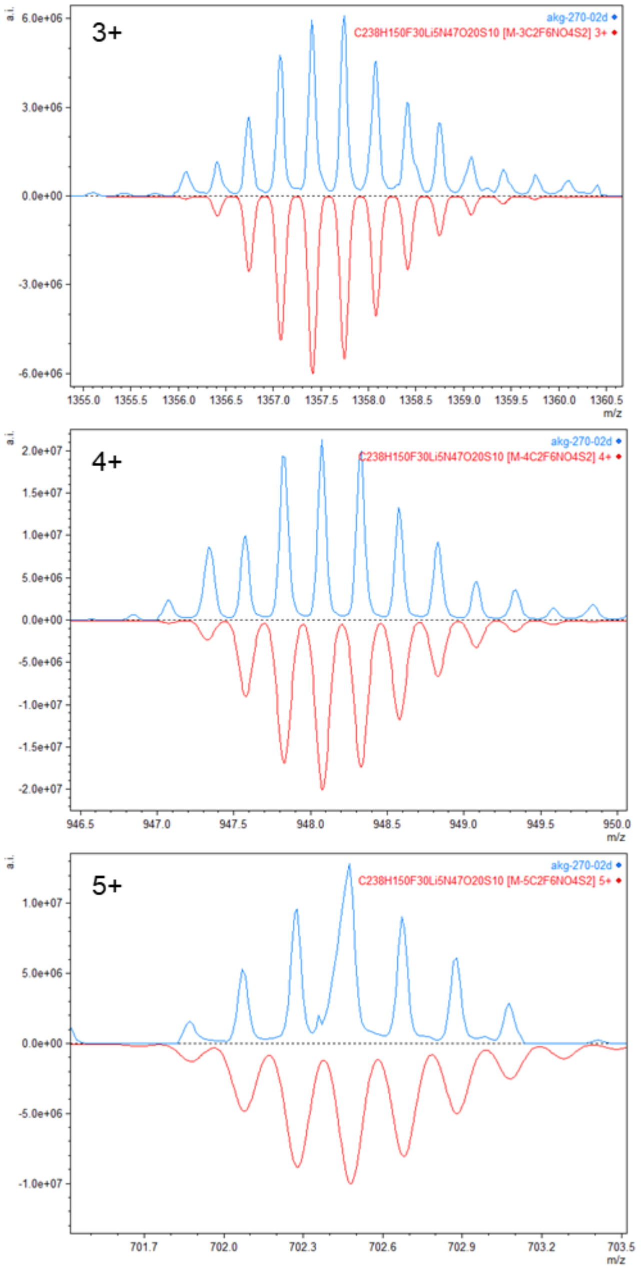

Figure S15. High-resolution ESI-mass spectrometry analysis of **1**·$(NTf_2)_5$ showing the observed (top blue) and theoretical (bottom red) isotope patterns for the +3, +4, and +5 peaks.

a)

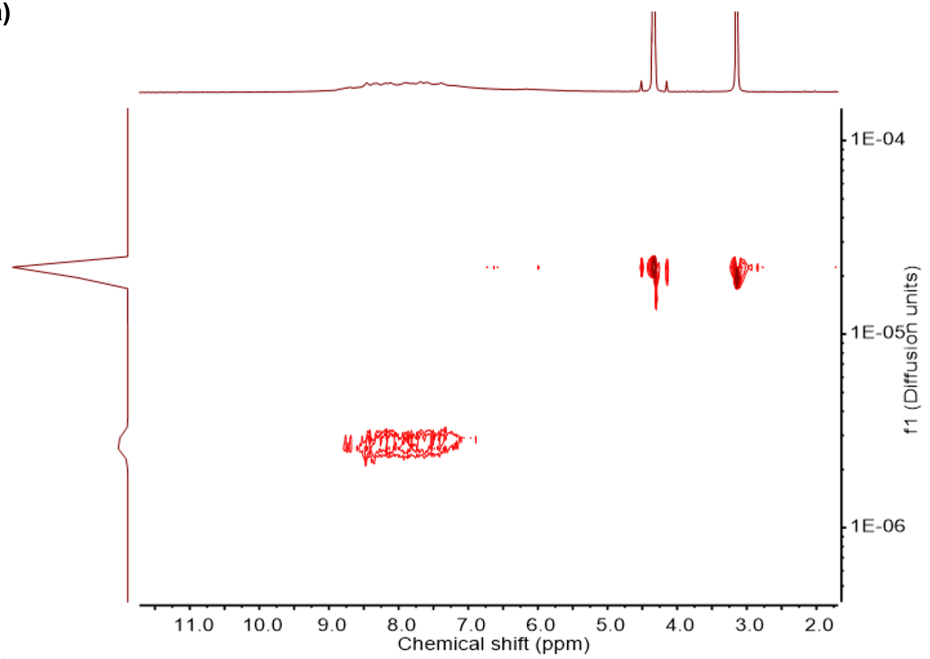

b)

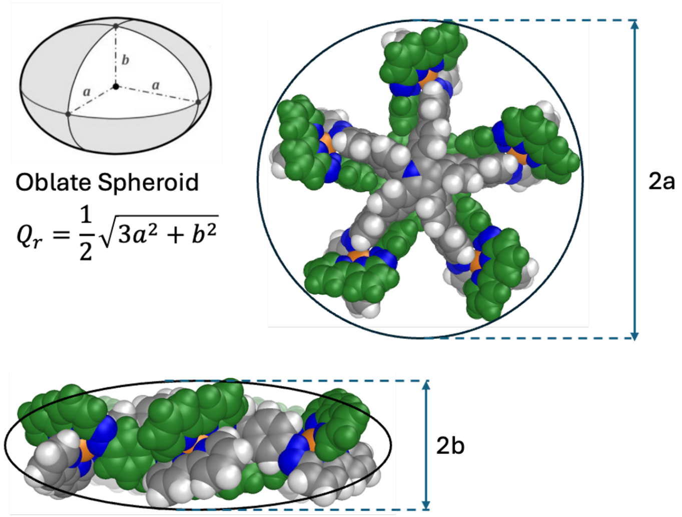

Figure S16. a) ^{1}H DOSY spectrum (400 MHz, 298 K, CD_3NO_2) of **1**. The diffusion coefficient for **1** in CD_3NO_2 was measured as 2.6×10^{-6} cm^2 s^{-1}, and the hydrodynamic radius was calculated to be 15.5 Å according to the Stokes-Einstein equation.[65] **b)** The ellipsoidal quadratic mean radius (Q_r) of a molecular model of **1**, optimized at the r2SCAN-3c level of theory (see SI Section 7.3), was estimated assuming an overall oblate spheroid shape.[66] The dimensions of a and b were estimated based on the average interatomic H–H distance as shown in the illustration, plus twice the van der Waals radius of H (1.09 Å). The estimated hydrodynamic radius was calculated to be 15.4 Å, consistent with the DOSY result obtained for **1**.

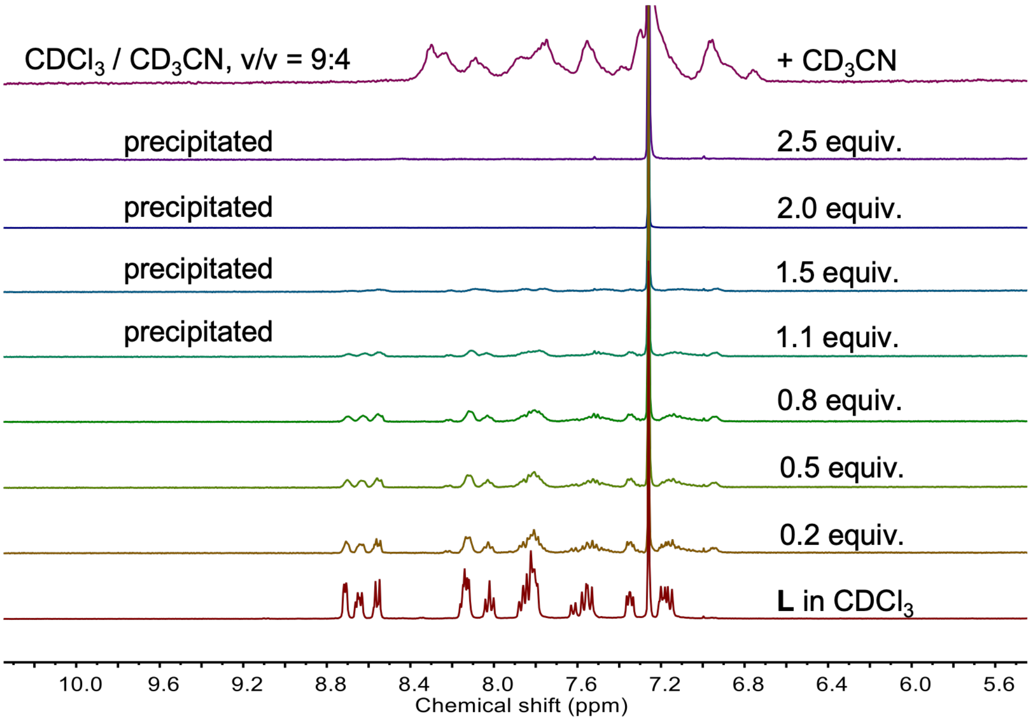

Figure S17. ^{1}H NMR titration (400 MHz, 298 K) of $LiNTf_2$ in CD_3CN (65.65 mM) into a $CDCl_3$ solution of **L** (0.73 mM) (equivalents of $LiNTf_2$ are labelled on the right). An orange precipitate was observed during the titration, indicating complex formation. The extra addition of CD_3CN ($CDCl_3$ / CD_3CN, v/v = 9:4) dissolved the precipitation. The HR-ESI mass spectrum confirmed the Li_5**L**$_2$ composition.

2.4 Self-assembly of **L’** with $LiNTf_2$

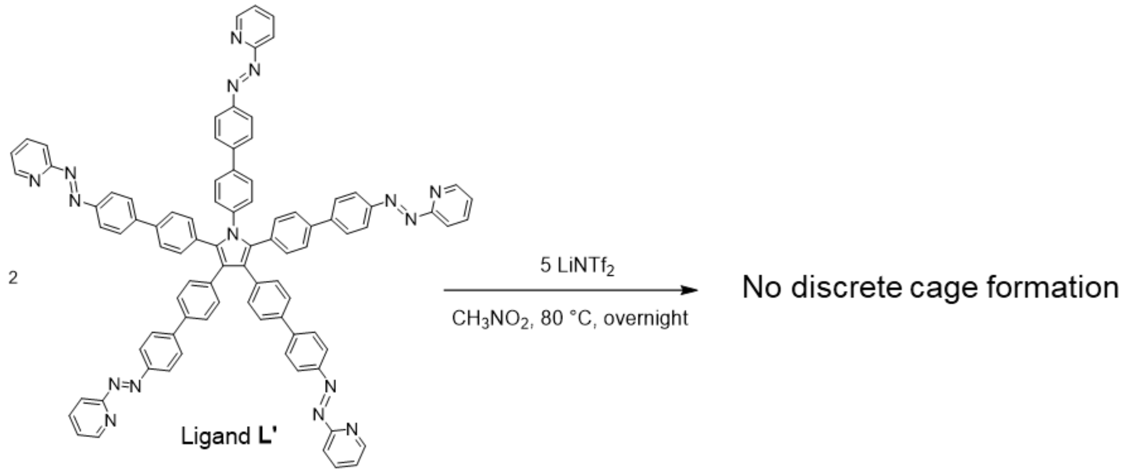

Scheme S4. Self-assembly of ligand **L’** with $LiNTf_2$.

Ligand **L’** (1.0 mg, 0.74 µmol, 1 equiv.), $LiNTf_2$ (0.53 mg, 1.85 µmol, 2.5 equiv.) and CH_3NO_2 (0.4 ml) were added to a vial, and the mixture was sonicated for 1 min. The mixture was then heated at 80 °C overnight. The solvent was removed by blowing with N_2 and then Et_2O (10 ml) was added. The resulting solid was collected by centrifugation, washed three times with additional Et_2O (10 ml) and then dried under dynamic vacuum for 16 h at 298 K The solid was then re-dissolved in CD_3NO_2 for NMR characterization.

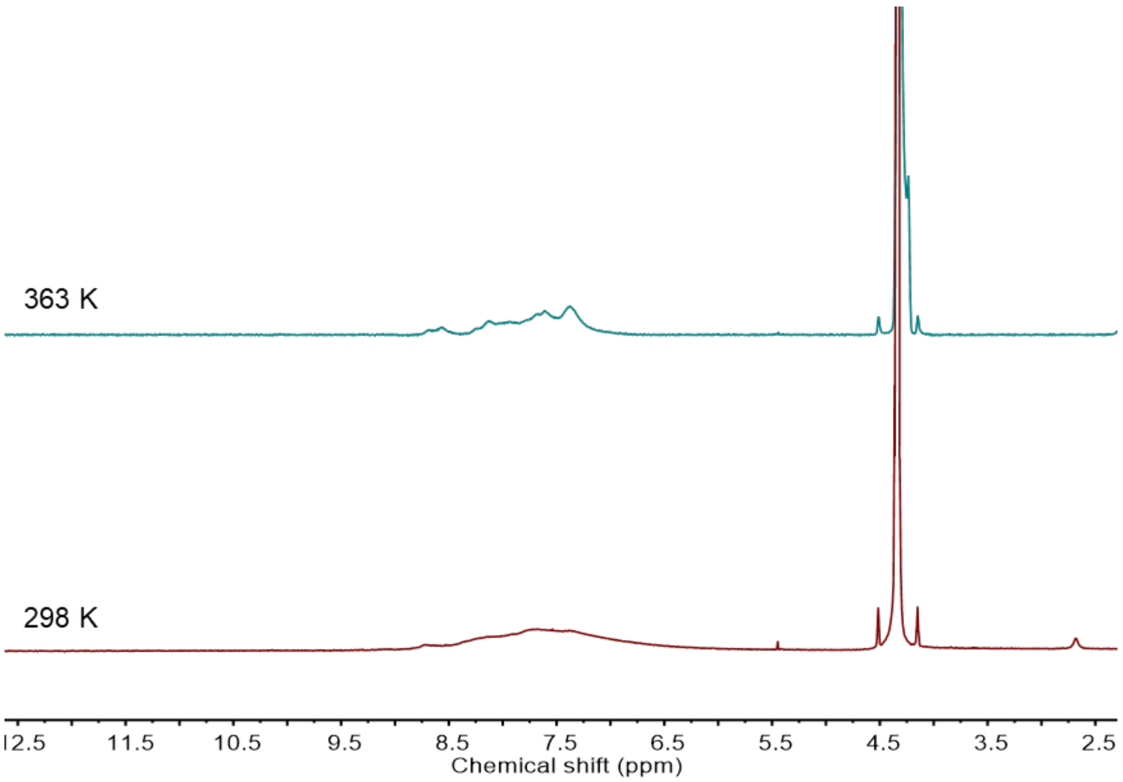

Figure S18. ^{1}H NMR (400 MHz, CD_3NO_2) of the **L'** + 2.5 $LiNTf_2$ sample above at 298 K and 363 K. Higher temperatures did not result in the emergence of identifiable peaks.

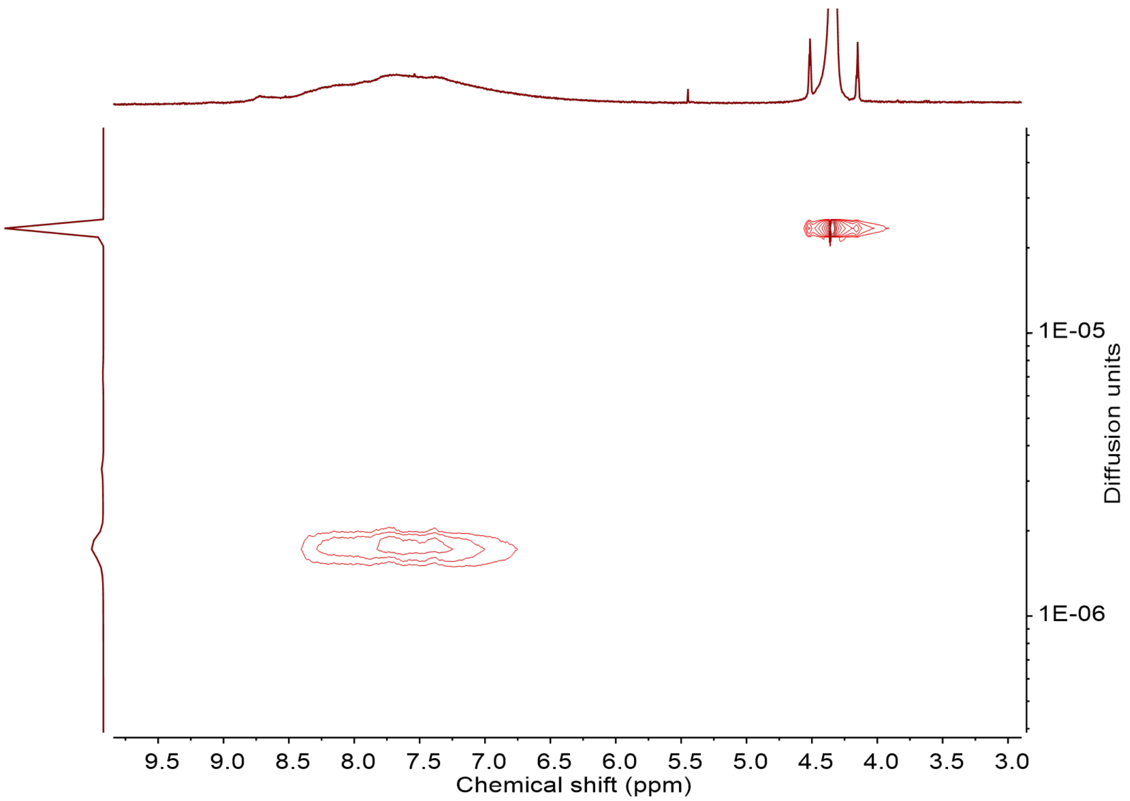

Figure S19. ^{1}H DOSY spectrum (400 MHz, 298 K, CD_3NO_2) of the **L'** + 2.5 $LiNTf_2$ sample. The diffusion coefficient in CD_3NO_2 was measured to be 1.7×10^{-6} cm^2 s^{-1}, and the hydrodynamic radius was calculated to be 24.0 Å according to the Stokes-Einstein equation.[65]

The hydrodynamic radius measured from DOSY was larger than the expected radius for a sandwich structure. In addition, no HR-ESI peaks corresponding to any discrete composition, such as Li_5**L'**$_2$, were observed.

2.5 Construction and characterization of the complex between **L** and $LiBF_4$

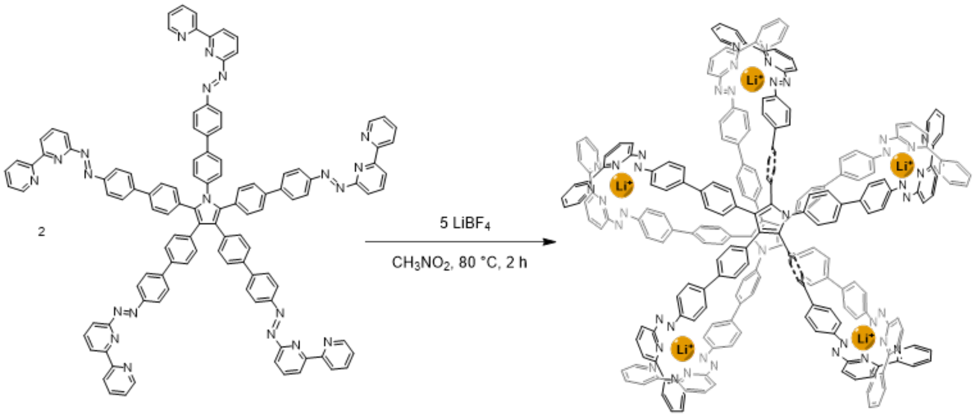

Scheme S5. Self-assembly of sandwich complex Li_5**L_2** (BF_4).

Ligand **L** (1.0 mg, 0.58 µmol, 1 equiv), $LiBF_4$ (0.13 mg, 1.45 µmol, 2.5 equiv) and CH_3NO_2 (0.4 ml) were added to a vial, and the mixture was sonicated for 1 min. The mixture was then heated to 80 °C for 2 hours. The solvent was removed by under a stream of N_2 and then Et_2O (10 ml) was added. The resulting solid was collected by centrifugation, washed three times with additional Et_2O (10 ml) and then dried under dynamic vacuum for 16 h at 298 K to give sandwich Li_5**L_2**$(BF_4)_5$ as a red solid (1.05 mg, 0.27 µmol, yield 93 %).

^{1}H NMR (400 MHz, CD_3NO_2) δ 9.03 (m, 10H), 8.90 – 8.86 (m, 20H), 8.56 (d, 10H), 8.42 (m, 10H), 8.25 (m, 10H), 8.17 – 8.12 (m, 30H), 7.99 (m, 20H), 7.83 (d, 4H), 7.74 (m, 16H), 7.48 (d, 4H), 7.41 (m, 16H).

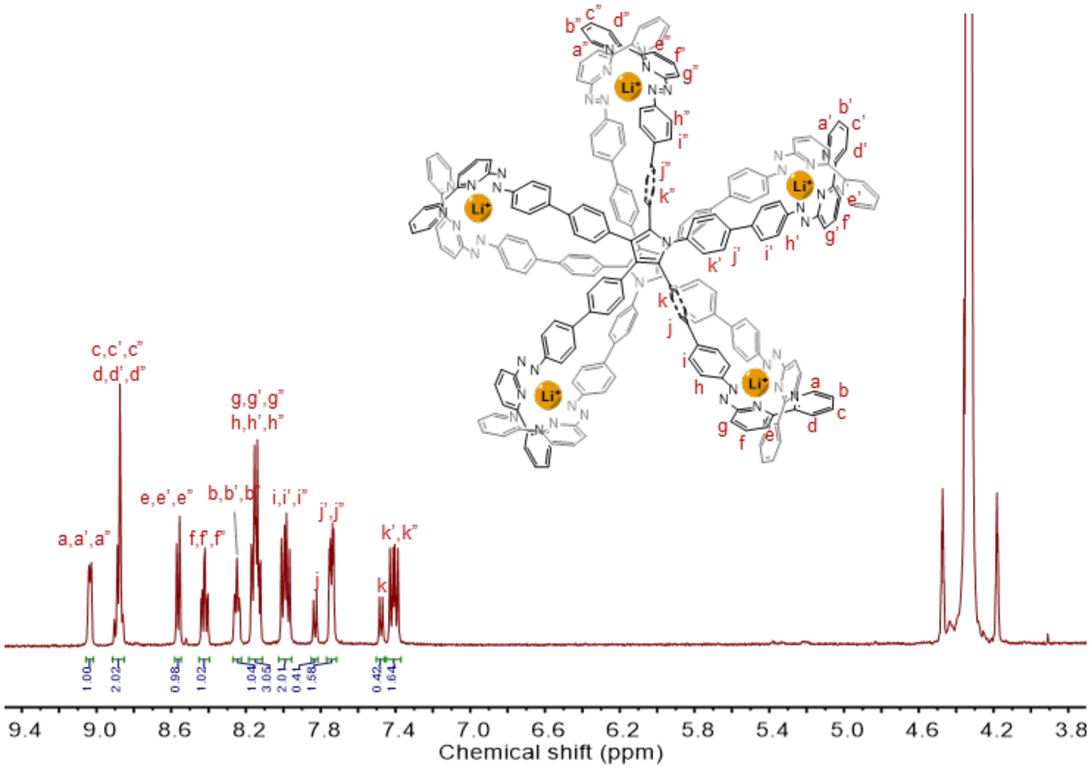

Figure S20. ^{1}H NMR spectrum (400 MHz, 298 K, CD_3NO_2) of $Li_5\mathbf{L}_2(BF_4)_5$.

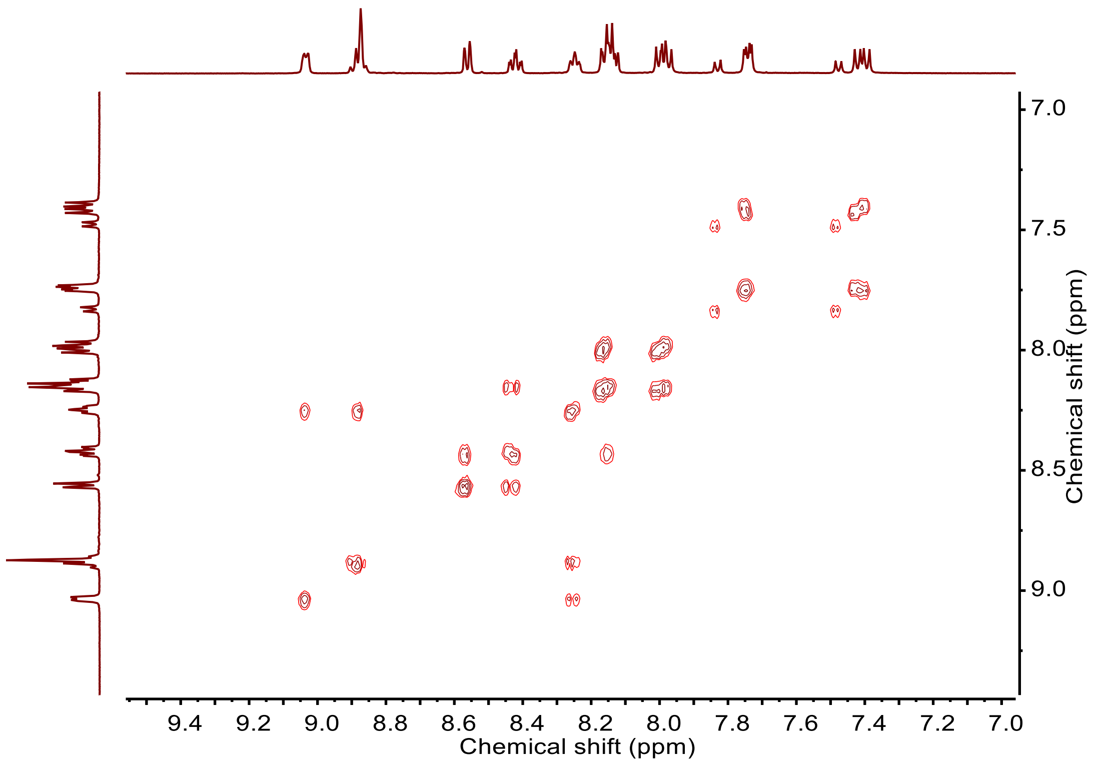

Figure S21. Partial ^{1}H-^{1}H COSY NMR spectrum (400 MHz, 298 K, CD_3NO_2) of $Li_5\mathbf{L}_2(BF_4)_5$.

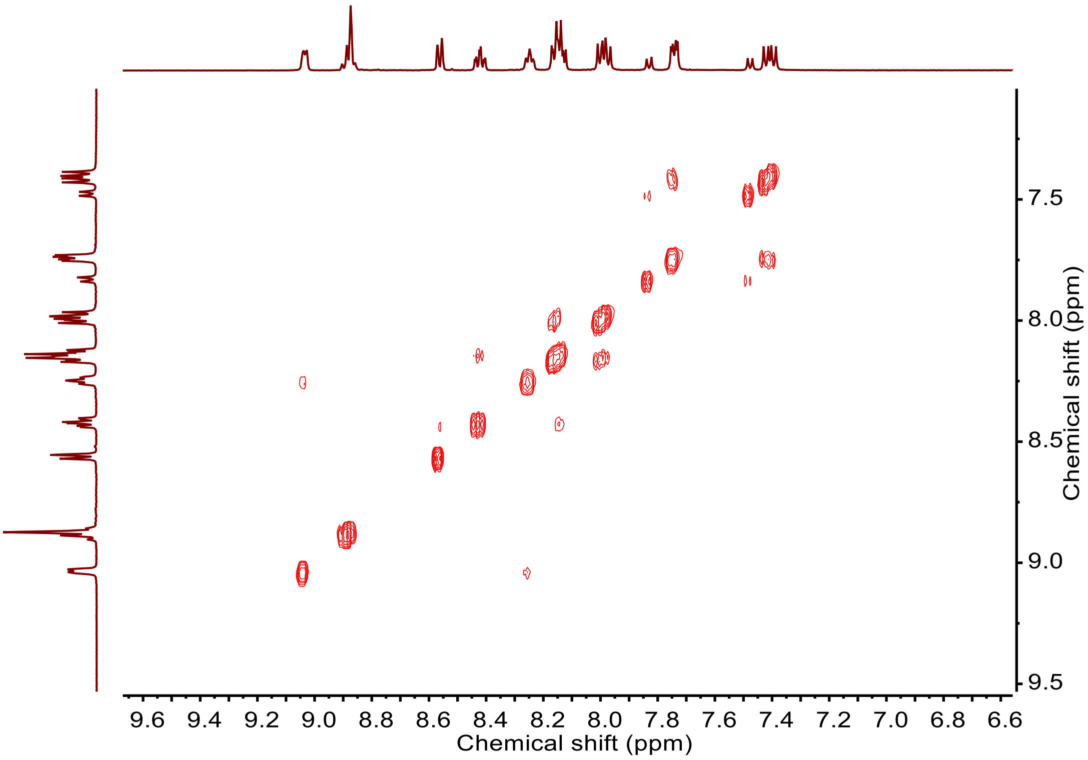

Figure S22. Partial ^{1}H-^{1}H NOESY NMR spectrum (400 MHz, 298 K, CD_3NO_2) of $Li_5\mathbf{L}_2(BF_4)_5$.

2.6 Self-assembly of iminopyridine-based analogs

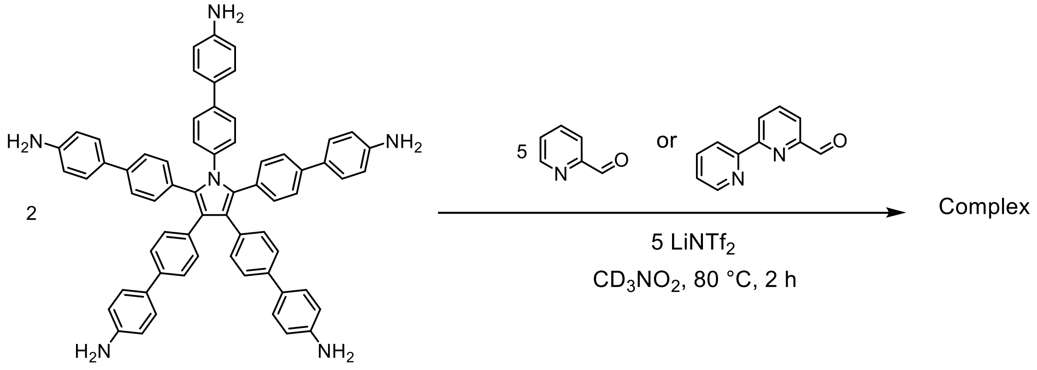

Compound **b**

Scheme S6. Self-assembly of iminopyridine-based complexes from compound **b**, 2-pyridinecarboxaldehyde or 6-formyl-2,2'-bipyridine, and $LiNTf_2$ in CD_3NO_2.

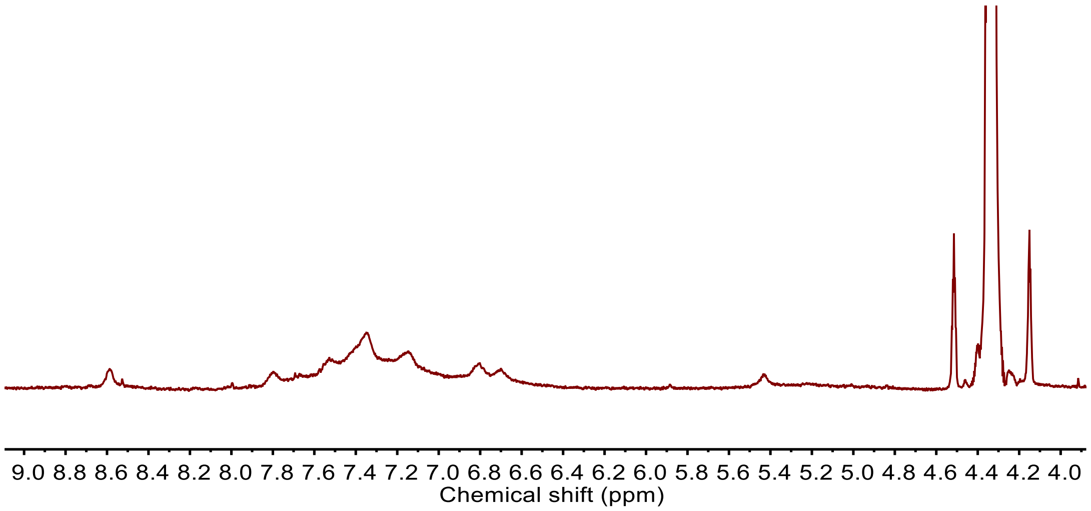

Figure S23. ^{1}H NMR spectrum (400 MHz, 298 K, CD_3NO_2) of the product formed from compound **b**, 2-formylpyridine and $LiNTf_2$.

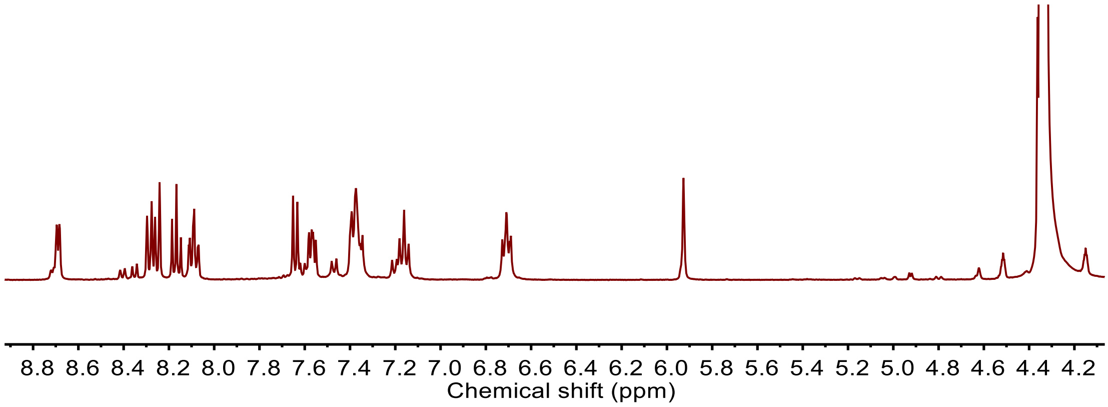

Figure S24. ^{1}H NMR spectrum (400 MHz, 298 K, CD_3NO_2) of the product formed from compound **b**, 6-formyl-2,2'-bipyridine and $LiNTf_2$.

3. Investigation of photoswitching properties

3.1 Photoswitching properties for ligand **L**

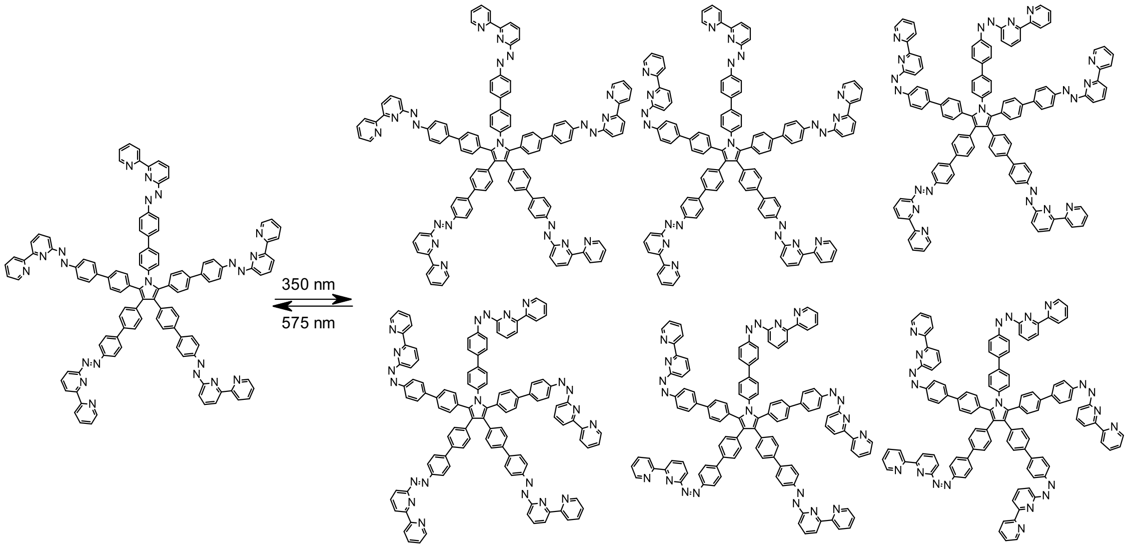

Figure S25. Reversible photoswitching of azobipyridine-containing ligand L**.** In an NMR tube, ligand **L** (1.00 mg, 0.57 μmol) was dissolved in 400 μL of $CDCl_3$. An NMR spectrum was measured immediately after irradiation at 350 nm for 15 min. To reverse the photoswitching, the same mixture was irradiated at 575 nm for 60 min.

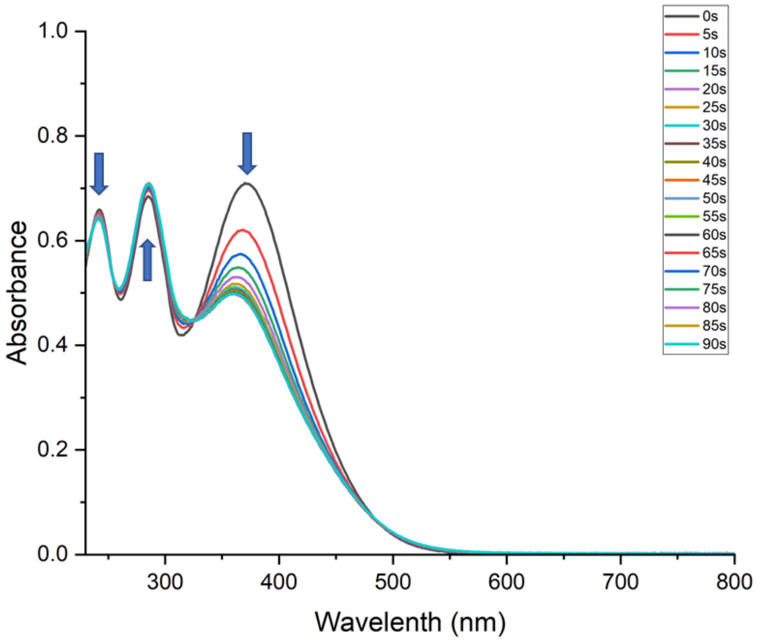

Figure S26. Time-dependent UV-vis spectrum of ligand **L** (49.8 μM, $CHCl_3$) under exposure to 350 nm light. The PSS was reached after 90 seconds.

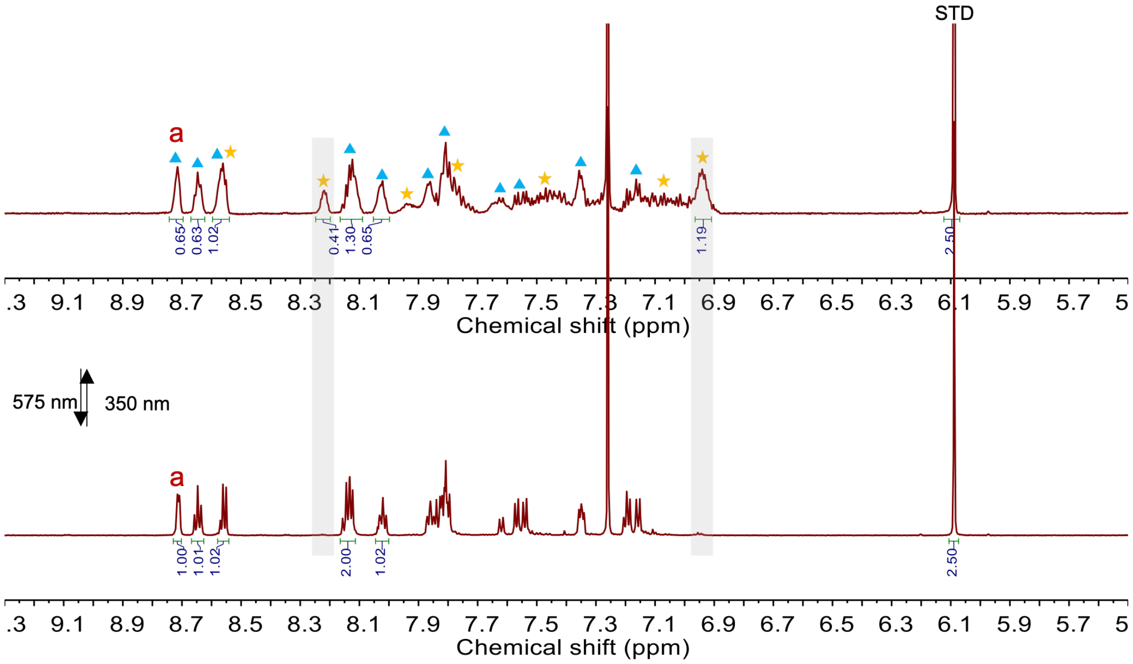

Figure S27. The comparison of ^{1}H NMR spectra (400 MHz, 298 K, $CDCl_3$) of **L** (4.3 mM) following exposure to light at 350 nm (15 min) and then at 575 nm (60 min) (400 MHz, $CDCl_3$, 25 °C). 1,3,5-Trimethoxybenzene was added as an internal standard. The protons of *trans*-**L** and *cis*-**L** are indicated by blue triangles and yellow stars, respectively. Due to the complexity of the spectra, it is difficult to assign peaks corresponding to *cis*-**L**. By comparing the integrals of proton a before and after irradiation at 350 nm, approximately 35% of *trans*-**L** is switched to *cis* form.

3.2 Photoswitching properties for complex **1**

Since the absorbance peak for CH_3NO_2 was found to overlap with that of complex **1**, we chose $CHCl_3$ as the solvent for the UV-Vis measurements. The complex was dissolved at a high concentration in CH_3NO_2 first (98.25 mM), followed by a 1500x dilution with $CHCl_3$ to minimize the effect of CH_3NO_2.

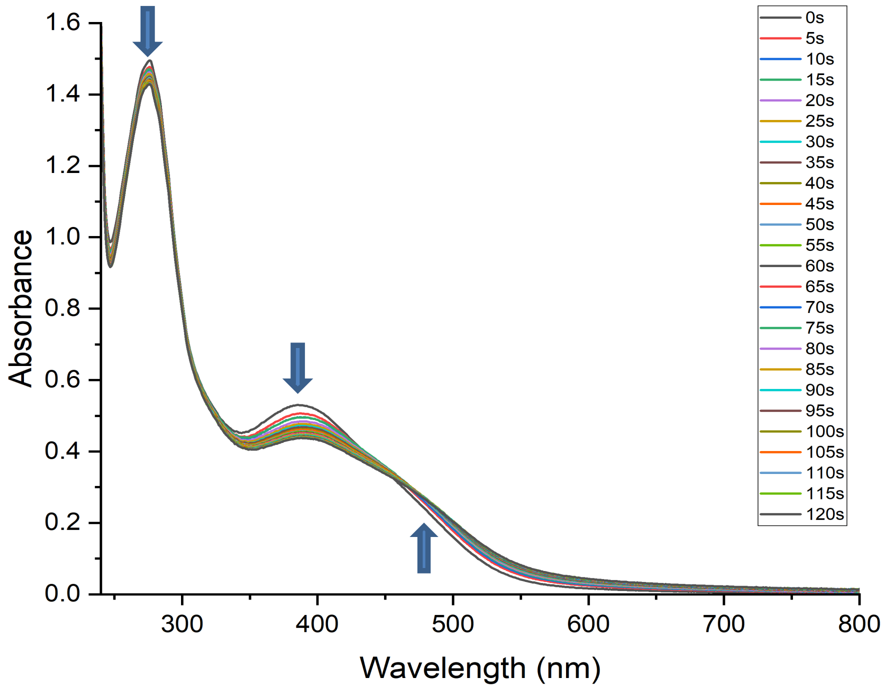

Figure S28. Time-dependent UV-vis spectrum of complex **1** (65.5 µM, $CHCl_3$) under the exposure of 350 nm light. The PSS was reached after 120 seconds.

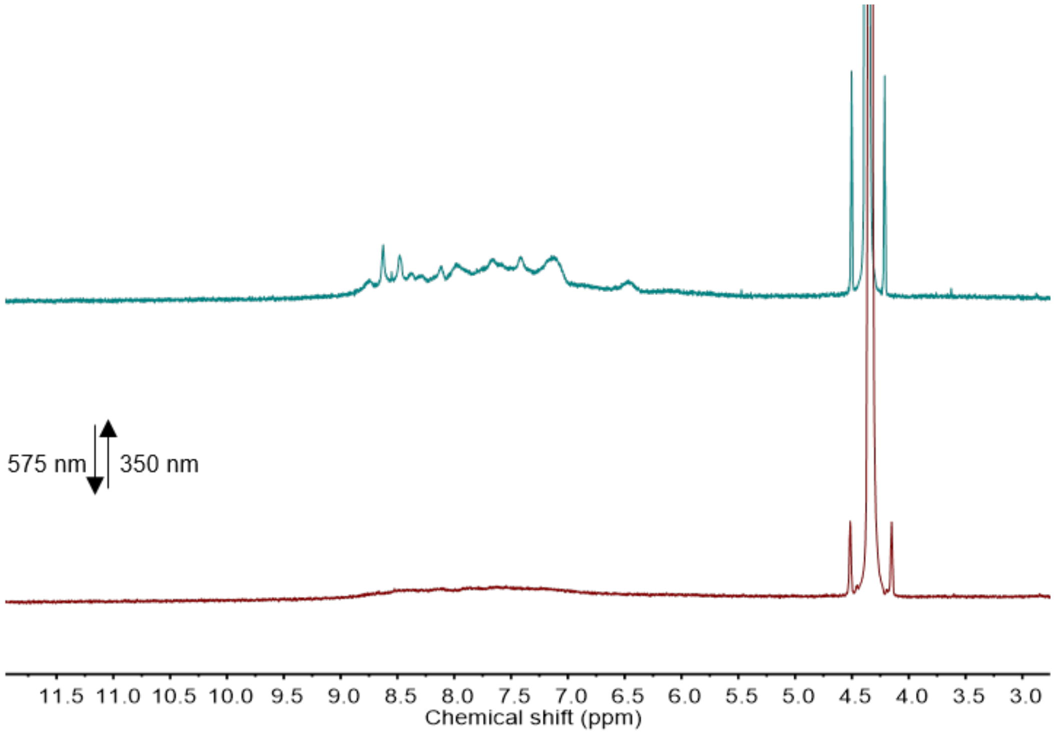

Figure S29. Comparison of ^{1}H NMR spectra (400 MHz, 298K, CD_3NO_2) of **1** (1.5 mM) following exposure to light at 350 nm (15 min) and then at 575 nm (60 min).

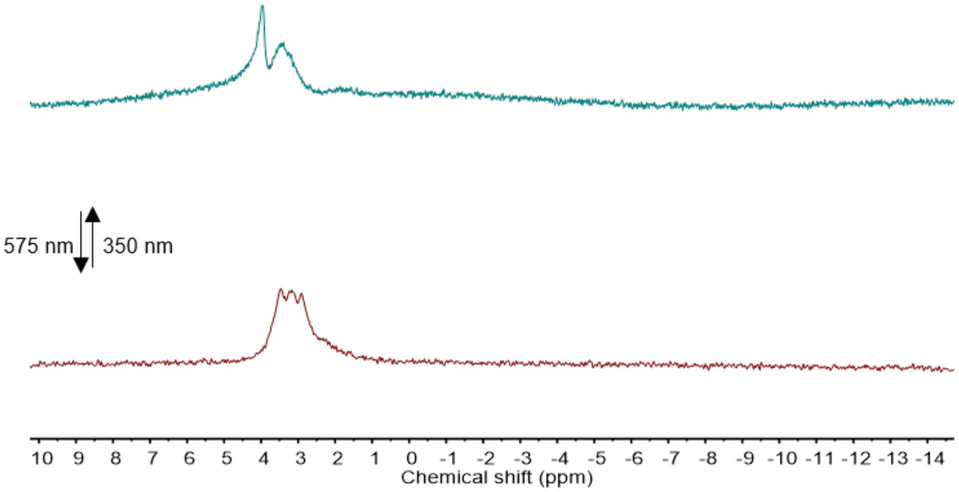

Figure S30. Comparison of ^{7}Li NMR spectra (194 MHz, 298K, CD_3NO_2) of **1** (1.5 mM) following exposure to light at 350 nm (15 min) and then at 575 nm (60 min).

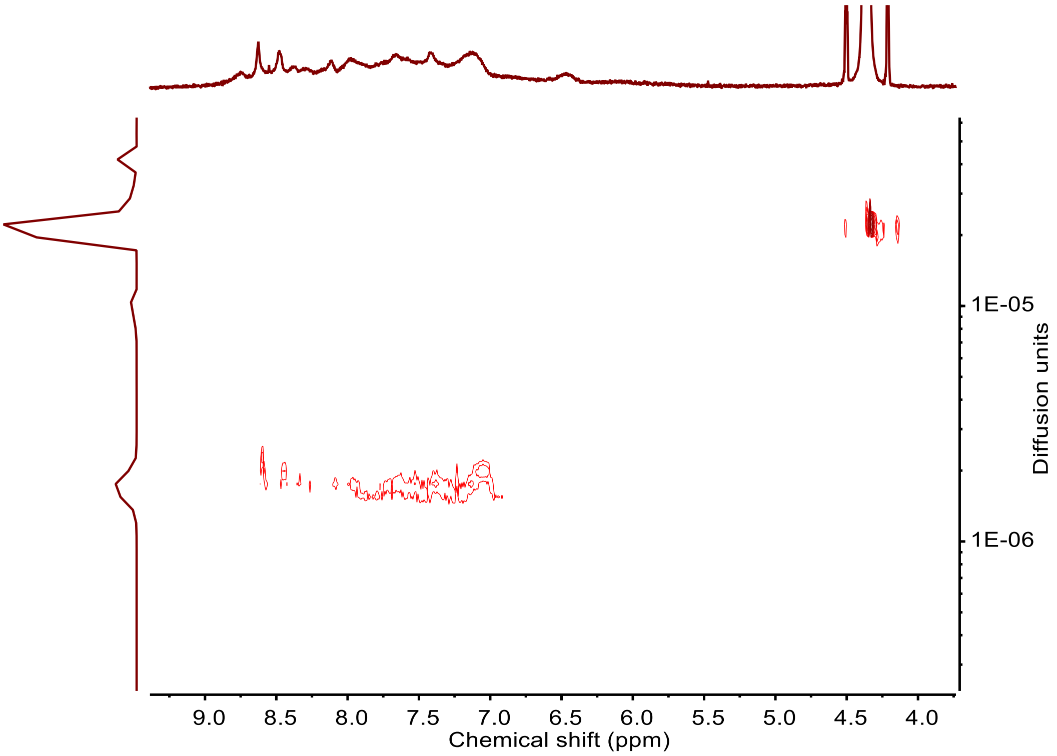

Figure S31. ^{1}H DOSY spectrum (400 MHz, 298 K, CD_3NO_2) of complex **1** after irradiation at 350 nm for 15 min, the diffusion coefficient in CD_3NO_2 was measured as 1.6×10^{-6} cm^2 s^{-1}, and the hydrodynamic radius was calculated to be 24.9 Å according to the Stokes-Einstein equation. The hydrodynamic radius was much larger than that before irradiation (15.5 Å). We infer that after irradiation at 350 nm, lithium is partially dissociated from the azobipyridine core, causing an increase in the hydrodynamic radius (See Figure 2).

4. Binding affinities of Li^+/K^+/Na^+ towards ligand L

The binding affinity of Li^+/K^+/Na^+ for ligand L was investigated by UV-Vis titration in a 10 mm UV-Vis cuvette. Since the absorbance region of CH_3NO_2 overlaps with that of ligand **L** and complex **1**, the titration was carried out in a $CHCl_3$/CH_3CN (v/v = 9:4) mixture. A solution of **L** in $CHCl_3$/CH_3CN (8.24×10^{-6} M) was transferred to a 10 mm cuvette and $LiNTf_2$/$NaNTf_2$/$KNTf_2$ (2.47×10^{-2} M) in $CHCl_3$/CH_3CN was gradually added. The solution in the cuvette was stirred for 2 minutes with a stir bar after each addition, followed by UV-Vis measurement. Concentration deviations due to dilution have been corrected for using calculated binding constants. The titration data were fitted using a Hill plot:

$$log\frac{\theta}{1-\theta} = nlog[Li] + \log K_a$$

where θ is the fraction of ligand bound by the lithium, which is determined by the observed change in absorbance ($\Delta\delta$) against the maximum absorbance change during titrations ($\Delta\delta_{max}$), n is the Hill coefficient describing cooperativity, and K_a is the apparent association constant.

$$K_a = \frac{[Li_5L_2]}{[Li]^5[L]^2}$$

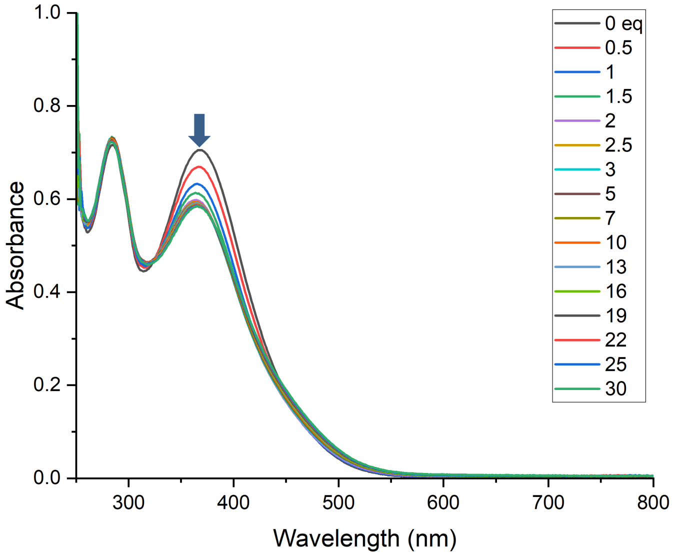

Figure S32. UV-Vis titration of $LiNTf_2$ into a solution of **L** in in $CHCl_3/CH_3CN$ (v/v = 9:4) at 298 K.

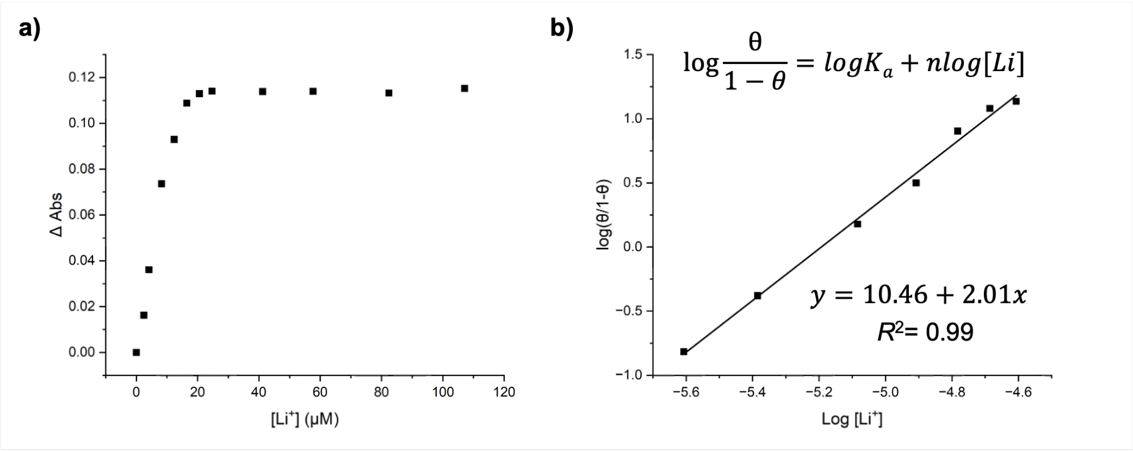

Figure S33. Titration curves fitted using the Hill function. (a) Δ Abs vs [Li^+] and (b) log[θ/(1 − θ)] vs log[Li^+]. The Hill coefficient was determined to be 2.01, indicating a positively cooperative binding mode. K_a was determined to be 2.88×10^{10} (mol $dm^{-3})^{-6}$. The positive cooperativity indicates that the binding of the first Li^+ promoted the preorganization of two ligands for the sandwich structure, so binding of subsequent Li^+ becomes progressively easier.

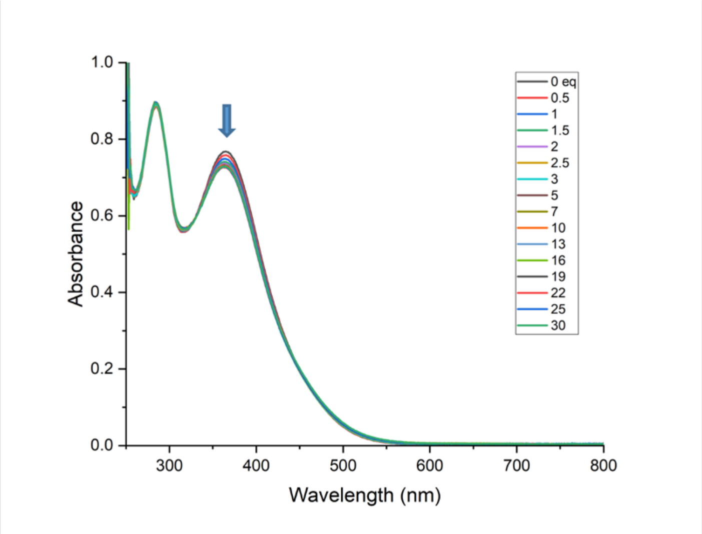

Figure S34. UV-Vis titration of $NaNTf_2$ into a solution of **L** in in $CHCl_3/CH_3CN$ (v/v = 9:4) at 298 K.

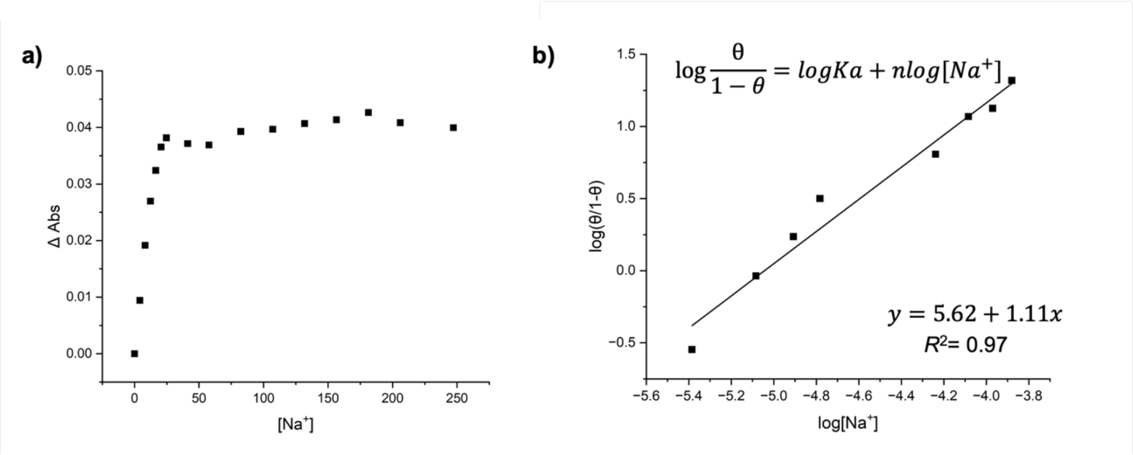

Figure S35. Titration curves fitted using the Hill function. (a) Δ Abs vs $[Na^+]$ and (b) $\log[\theta/(1-\theta)]$ vs $\log[Na^+]$. The Hill coefficient was determined to be 1.14, indicating a positively cooperative binding mode. The K_a was determined to be 4.16×10^5 (mol $dm^{-3})^{-6}$. Note that the K_a value is apparently large due to the units being different from systems with a simple 1:1 or 1:2 binding mode. This K_a was 10^5 times smaller than the K_a value obtained for Li^+, using the same method.

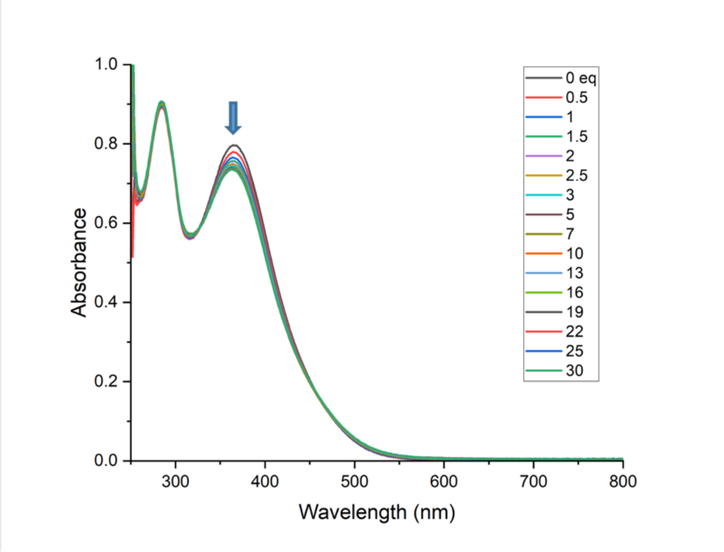

Figure S36. UV-Vis titration of $KNTf_2$ into a solution of **L** in in $CHCl_3/CH_3CN$ (v/v = 9:4) at 298 K.

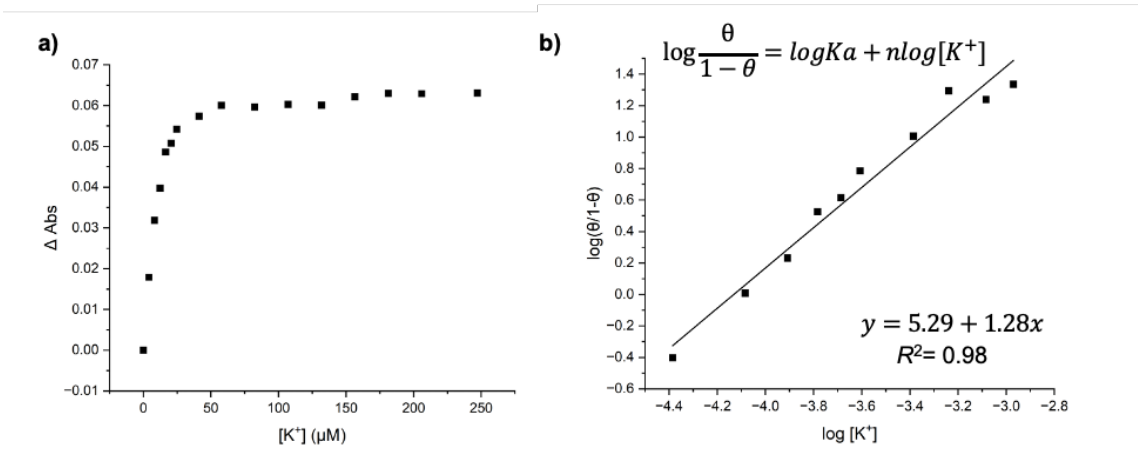

Figure S37. Titration curves fitted using the Hill function. (a) Δ Abs vs $[K^+]$ and (b) $\log[\theta/(1 - \theta)]$ vs $\log[K^+]$. The Hill coefficient was determined to be 1.28, indicating a positively cooperative binding mode. The K_a was determined to be 1.95×10^5 (mol $dm^{-3})^{-6}$. Note that the K_a value is apparently large due to the units being different from systems with a simple 1:1 or 1:2 binding mode. This K_a was 10^5 times smaller than the K_a value obtained for Li^+, using the same method.

5. Selectivity of alkali metal ions

Ligand **L** (1.0 mg, 0.58 µmol, 1 equiv.), $LiNTf_2$ (0.42 mg, 1.45 µmol, 2.5 equiv.) or $NaNTf_2$ (0.44 mg, 1.45 µmol, 2.5 equiv.) or $KNTf_2$ (0.46 mg, 1.45 µmol, 2.5 equiv.) were added to a vial, followed by the addition of 0.4 mL CH_3NO_2. The mixture was sonicated for 1 min, and then heated at 80 °C overnight. A soluble complex was only formed for the reaction mixture with $LiNTf_2$, whereas for Na^+ and K^+ dissolution was not observed to occur.

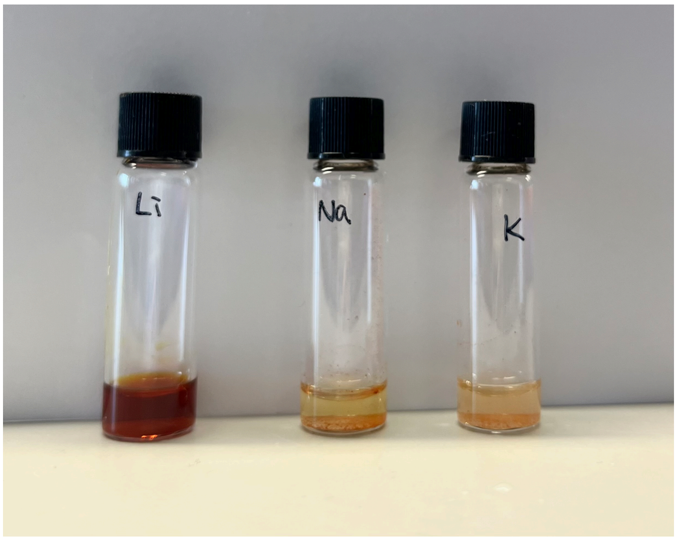

Figure S38. Photo of the three reaction mixtures involving 2:5 mixtures of **L** with the three alkali metal triflimide salts: Li^+ (left), Na^+ (middle) and K^+ (right), after heating at 80 °C overnight. Only the Li^+ reaction mixture exhibited a clear deep orange color without precipitate.

As described in Section 2.3, **1** could also be formed in a mixture of $CDCl_3$ / CD_3CN (v/v = 9:4). Therefore, we investigated whether a complex is formed for Na^+ and K^+ under these conditions. Ligand **L** (1.0 mg, 0.58 µmol, 1 equiv.) was dissolved in 520 µL $CDCl_3$ / CD_3CN (v/v = 9:4) solution. $NaNTf_2$ (0.44 mg, 1.45 µmol, 2.5 equiv.) or $KNTf_2$ (0.46 mg, 1.45 µmol, 2.5 equiv.) were added. The addition of a concentrated CD_3CN solution of $NaNTf_2$ or $KNTf_2$ into $CDCl_3$ solution of **L** ($CDCl_3$ / CD_3CN, v/v = 20:1) did not produce any precipitate, in contrast to $LiNTf_2$.

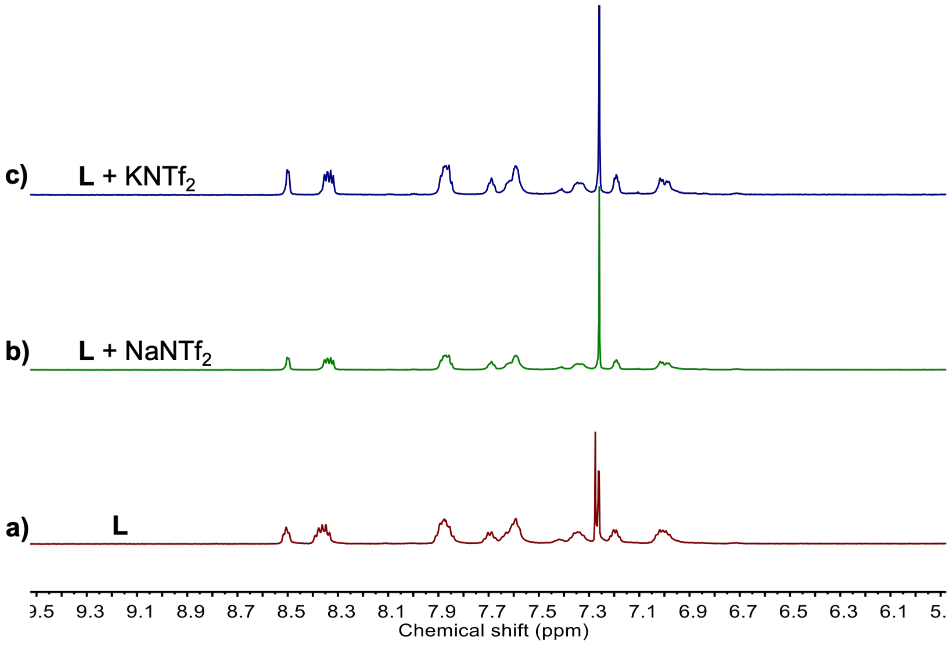

Figure S39. ^1H NMR spectra (500 MHz, 298 K, $CDCl_3$ / CD_3CN, v/v = 9:4) of **a) L**; **b) L**+$NaNTf_2$; c) **L**+$KNTf_2$. ^1H NMR chemical shifts showed no significant changes after addition of the metal salts, indicating that Na^+ and K^+ have weak or no interaction with ligand **L**.

6. Lithium Extraction cycle

6.1 General procedures

Ligand **L** was first dissolved in $CHCl_3$ (2 mL, and an equimolar mixture of $NaNTf_2$, $LiNTf_2$ and $KNTf_2$ in CH_3CN (solution **a**) was then added. Note that the proportion of $CHCl_3$ and CH_3CN was controlled to be 20:1 to maximize the precipitation of **1**. After decanting the supernatant, the precipitate was dissolved in CH_3NO_2. Demineralized water was then added, followed by illumination at 350 nm for 30 min. The free Li^+ went into the water phase and **L** precipitated. The addition of $CHCl_3$ and irradiation at 575 nm for 1 h then regenerated *trans*-**L**.

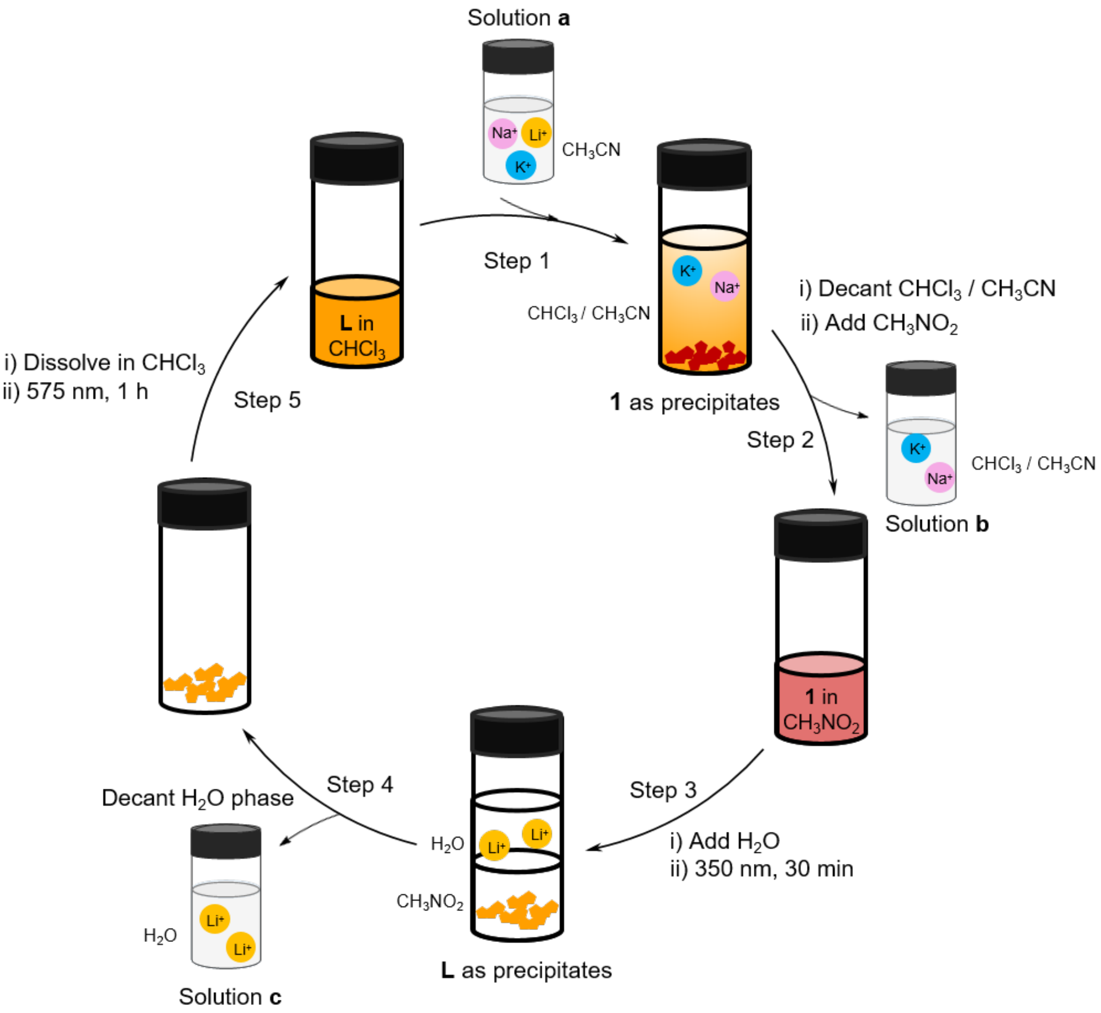

Figure S40. Illustration of lithium extraction from Na^+ and K^+, as shown in the main text as Figure 3.

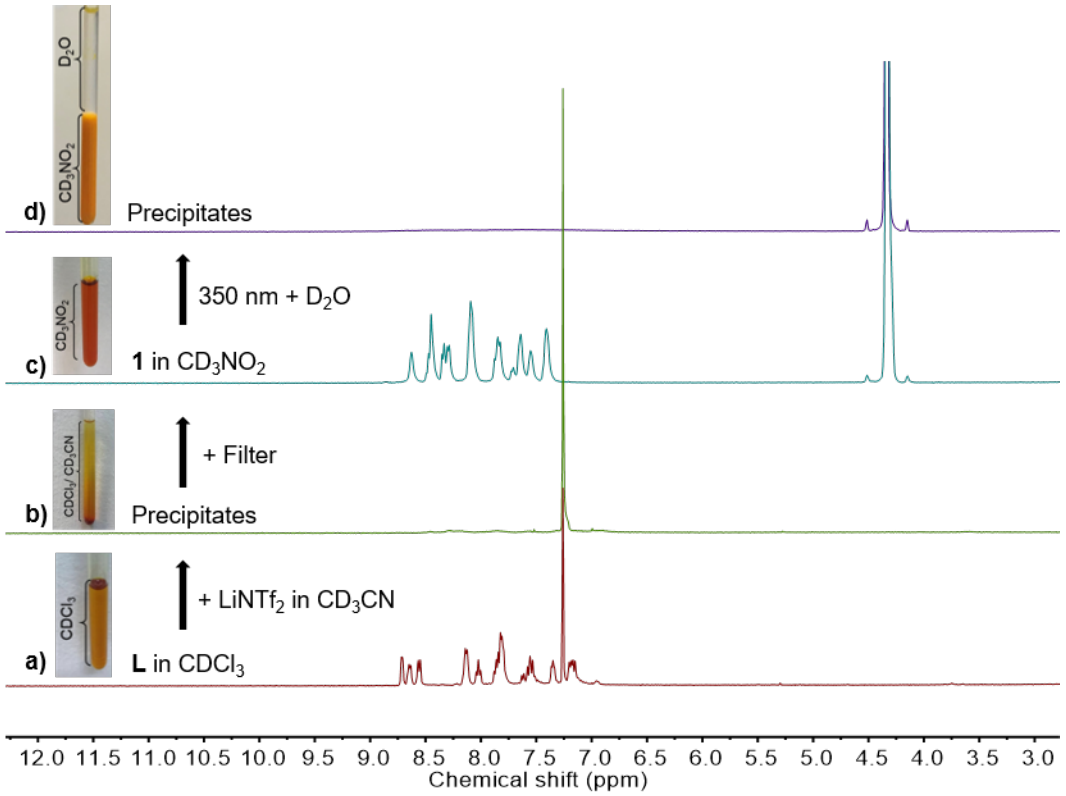

Figure 41. Comparison of ^{1}H NMR (400 MHz) spectra. a) L in 400 µL $CDCl_3$ (1.44 mM); **b)** after addition of 20 µL 71.5 mM $LiNTf_2$ in CD_3CN; **c)** The precipitate was then redissolved in 450 µL CD_3NO_2, and the spectrum was measured at 363 K; **d)** Complex **1** was then irradiated at 350 nm for 30 min and 450 µL D_2O was added. Spectra **a)**, **b)** and **d)** were measured at 298 K.

6.2 Inductively Coupled Plasma Spectrometry (ICP) Analysis

First, a 50 mL solution of 0.32 M HNO_3 was prepared, from which 10 mL was taken as a reference for ICP measurements. Next, a mixture of $KNTf_2$ (3.99 mg, 12.50 µmol), $NaNTf_2$ (3.79 mg, 12.50 µmol), and $LiNTf_2$ (3.59 mg, 12.50 µmol) was dissolved in 500 µL of CH_3CN. From this mixture, 100 µL was extracted and placed in a vial. The CH_3CN solvent was evaporated, and the remaining solid was dissolved in 10 mL of the 0.32 M HNO_3 solution, which is referred to as solution **a** in Figure S38. This solution was then submitted for ICP analysis.

Ligand **L** (1.74 mg, 1.00 µmol) was dissolved in 2 mL of $CHCl_3$. Then, 100 µL of the

CH_3CN solution containing Na^+, K^+, and Li^+ was added. Ligand **L** immediately formed a $Li_5\mathbf{L}_2$ structure **1**, which precipitated out as a red solid, while Na^+ and K^+ remained in solution. The solution was then filtered through a glass-fiber filter in a pipette to remove the precipitate, and the filtrate was collected. The filtrate was evaporated, and the residue was dissolved in 10 mL of the 0.32 M HNO_3 solution, which is referred to as solution **b** in Figure S30. This solution was then submitted for ICP analysis.

The separated red solid precipitate, corresponding to **1**, was then dissolved in 1 mL of nitromethane. To this red solution, 1 mL of water was added, and the mixture was shaken and allowed to sit for 15 minutes. This biphasic solution was then irradiated with 350 nm light for 30 minutes and shaken. Yellow solids were observed in the nitromethane phase, while the water phase remained clear. The water layer was separated and evaporated, and the residue was dissolved in 10 mL of the 0.32 M HNO_3 solution, which is referred to as solution **c** in Figure S38. ICP analysis was performed on this solution (Figure S40 and table S1).

The nitromethane phase was filtered, and the resulting yellow precipitate was dried and dissolved in $CHCl_3$. The solution was irradiated with 575 nm light to generate the free ligand **L**, which was then recycled.

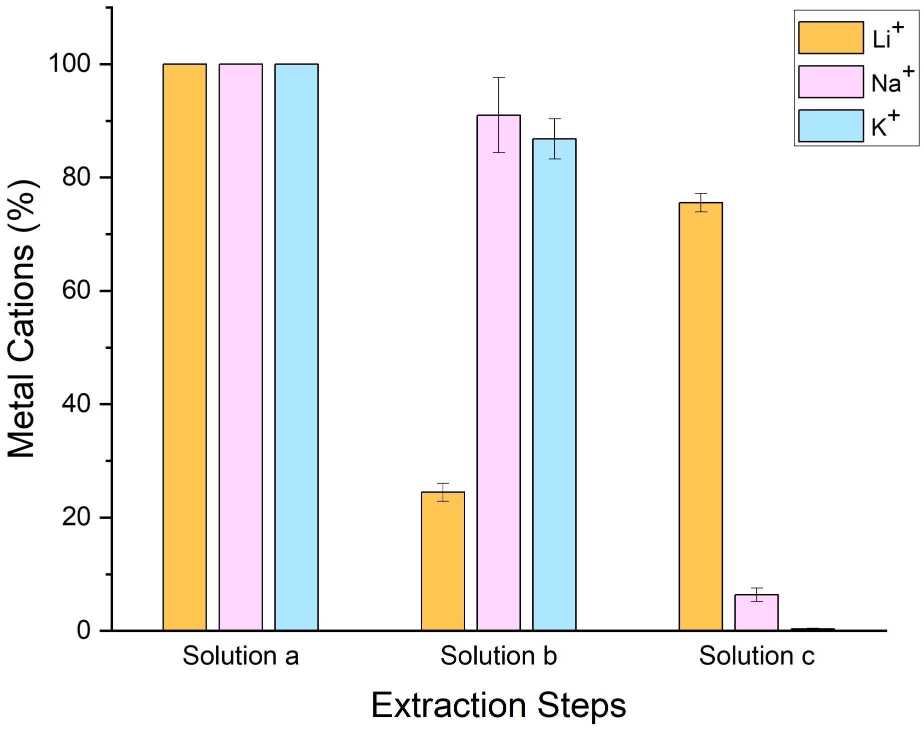

Figure S42. The concentrations of Na^{+}, K^{+}, and Li^{+} were measured at each extraction step using ICP analysis. In the first step, the concentration values obtained from ICP for each metal cation were normalized to a scale of 100. The highest observed concentration was set thus to 100, and the concentrations in the second and third steps were normalized accordingly.

Metal	Solution a	Solution b	Solution c
Li	100%	24% (±1.6%)	75% (±1.6%)
Na	100%	91% (±6.6%)	6% (±1.2%)
K	100%	87% (±3.5%)	0.3% (±0.1%)

Table S1. The results of ICP analysis as shown in Figure S32.

6.3 Stability over five rounds recycling.

To test the recyclability of ligand **L** over multiple rounds of lithium extraction, we performed ^{1}H NMR using 1,3,5-methoxybezene as the internal standard. We first prepared a stock solution of internal standard in $CDCl_3$ (60 mM). We then performed the extraction cycle as described above in Section 6.1. We measured ^{1}H NMR spectra at the beginning and the end of every extraction cycle, adding the same amount of internal standard.

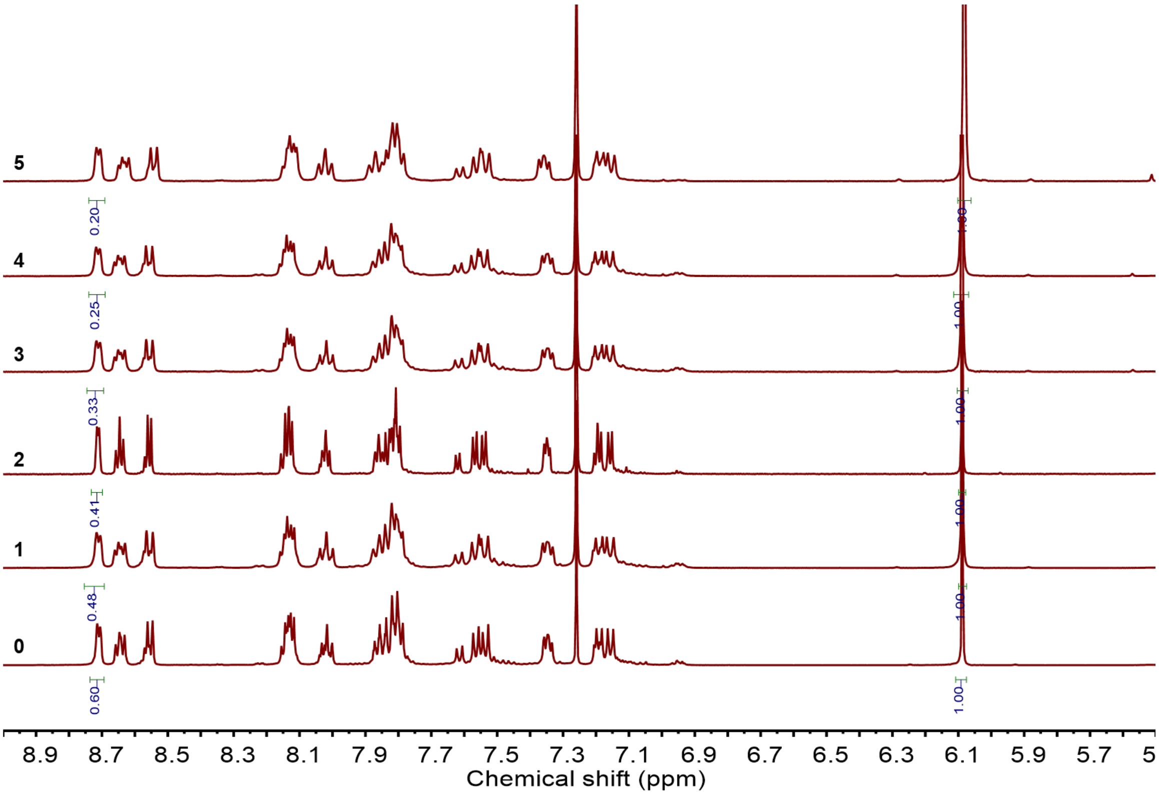

Figure 43. Comparison of ^{1}H NMR (400 MHz, 298 K, $CDCl_3$) spectra containing **L** and internal 1,3,5-methoxybenzene standard after five cycles. 80% was recovered after the first cycle and 33.3% was recovered after five cycles.

7. Geometry optimization and energy evaluations of sandwich complex 1

7.1 Enumeration of pentagonal sandwich diastereomers

The number of distinct pentagonal sandwich structures was counted using Burnside's lemma:

Let G be a group acting on a finite set X.

For any $g \in G$, we define X^g as the set of elements of X fixed by g, so $X^g = \{x \in X | g(x) = x\}$.

Then the number of orbits ($|X/G|$) is given by the average number of elements fixed by G:

$$|X/G| = \frac{1}{|G|} \sum_{g \in G} |X^g|$$

This lemma[67] uses group theory to count the total number of distinct diastereomers, or in other words, calculate the number of 'orbits' ($|X/G|$) for finite set X. In this case, X is the set of 2^5 possible vertex stereochemistries (Δ/Λ) and 5^2 possible linker orientations (N-orientations) that can be chosen for a pentagonal sandwich with pyrrole-based linkers, leading to 800 possible ways to choose the faces and vertices when excluding any symmetry considerations. Two combinations are considered the same when they are related by a symmetry element (g). For example, two combinations are identical when one of them can be obtained by simply rotating the other. The relevant symmetry elements are defined by the symmetry (point) group G of the set X. For the pentagonal sandwich (ignoring any chemistry) this will be the D_{5h} symmetry group (G = D_{5h}). For each of the symmetry elements g in the group G, the number of permutations that are fixed by this symmetry element are counted ($|X^g|$). A permutation x is fixed by g if it remains unchanged when applying this symmetry element on it ($g \cdot x = x$). The values $|X^g|$ are then added together to yield the total number of distinct permutations or 'orbits'.

There are 20 symmetry elements defined in the character table for point group D_{5h} ($|G| = 20$). The first 10 elements for D_5 are rotational (or orientation-preserving) symmetries consisting of the identity element E, two 72° face rotations ($2C_5$), two 144° face rotations ($2C_5^2$) and five 180° edge rotations ($5C'_2$). The remaining 10 symmetry elements are one mirror plane (σ_h), two 72° improper rotation axes ($2S_5$), two 216° improper rotation axes ($2S_5^3$, see equation 1) and five vertical reflection planes ($5\sigma_v$).

An improper rotation axis consists of a rotation around an axis and a reflection perpendicular to that axis. When enantiomers are distinguished from each other, the symmetry elements including reflections should be ignored and only rotational symmetries should be counted, which is consistent with point group D_5 ($|G|$=10). Both types of calculations will be described in this section. The number of unique diastereomers that are achiral can be obtained with equations 2-4.

$$S_5^3 = (C_5 \cdot \sigma_h) \cdot (C_5 \cdot \sigma_h) \cdot (C_5 \cdot \sigma_h) \quad \text{(Eq. 1)}$$

$$\left|\frac{X}{D_{5h}}\right| = n_{chiral} + n_{achiral} \quad \text{(Eq. 2)}$$

$$n_{chiral} = \left|\frac{X}{D_5}\right| - \left|\frac{X}{D_{5h}}\right| \quad \text{(Eq. 3)}$$

$$n_{achiral} = \left|\frac{X}{D_{5h}}\right| - n_{chiral} = \left|\frac{X}{D_{5h}}\right| - \left(\left|\frac{X}{D_5}\right| - \left|\frac{X}{D_{3h}}\right|\right) = 2\left|\frac{X}{D_{5h}}\right| - \left|\frac{X}{D_5}\right| \quad \text{(Eq. 4)}$$

Ligand arrangements

The first calculation that will be discussed is that of the number of unique ways to choose the orientations of two pentagonal faces, where vertex stereochemistry is ignored (equations 5 and 6). Figure S44 illustrates the effect of each symmetry element on picking a particular ligand orientation. The identity element E does not fix any faces; therefore, all linker orientations can be picked independently. The number of fixed permutations for E will be the total number of combinations possible for the faces ($|X^E|_F = 5^2$). The linkers are not symmetric with respect to the C_5 operations and these terms cancel out ($|X^{C_5}|_F = \left|X^{C_5^2}\right|_F = 0$). The linker is symmetric with respect to the C_2' rotation and σ_h mirror plane, where both fix a face when picking the other ($\left|X^{C_2'}\right|_F = |X^{\sigma_h}|_F = 5^1$). The linker is again not symmetric with respect to the improper rotation axes ($|X^{S_5}|_F = \left|X^{S_5^3}\right|_F = 0$). Only one linker orientation is allowed for each face when adhering to the vertical mirror plane. ($|X^{\sigma_v}|_F = 1^2$). From this result we conclude that there are three unique linker arrangements, where one is achiral (α) and two are chiral (β and γ).

$$\left|\frac{X}{D_5}\right|_F = \frac{1}{10}(5^2 + 2\cdot 0 + 2\cdot 0 + 5\cdot 5^1) = 5 \quad \text{(Eq. 5)}$$

$$\left|\frac{X}{D_{5h}}\right|_F = \frac{1}{20}(5^2 + 2\cdot 0 + 2\cdot 0 + 5\cdot 1^2 + 5^1 + 2\cdot 0 + 2\cdot 0 + 5\cdot 1^2) = 3 \quad \text{(Eq. 6)}$$

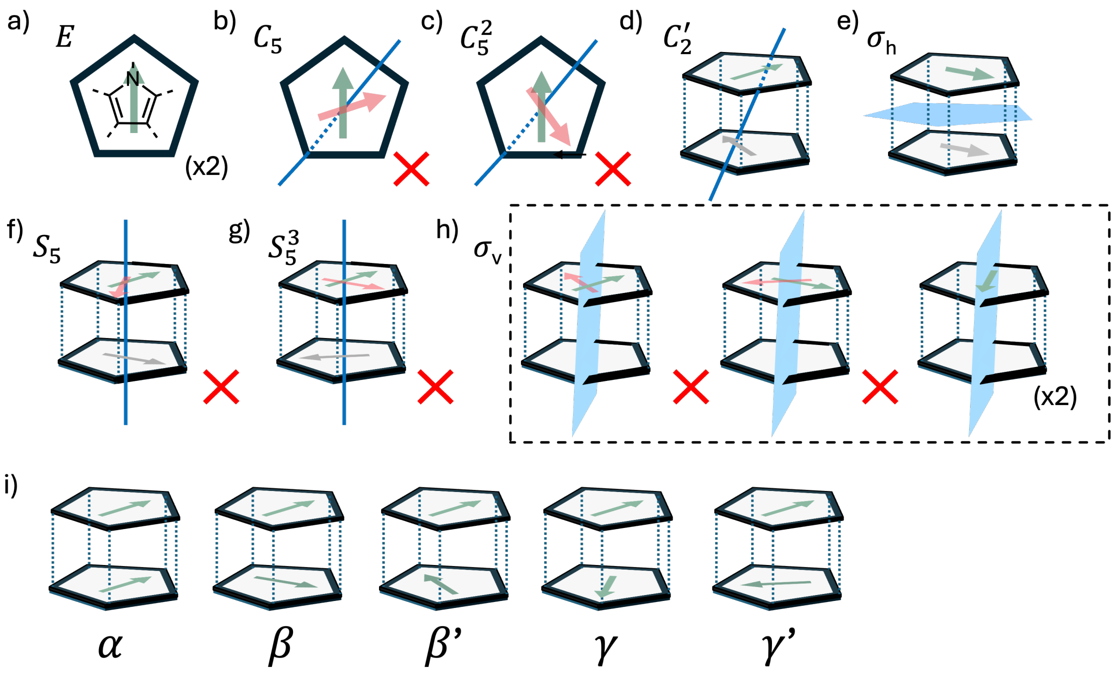

Figure S44. Description of the number of pentagonal faces that are fixed by a symmetry element (grey arrow) upon picking the orientation of one face (green arrow). A red arrow and red cross are shown when a linker is not symmetric with that symmetry element. The (x2) note is added when both faces can be picked independently from each other, resulting in a squared term in the equation.

Vertex stereochemistry

In the next case, the ligand arrangements are ignored and only the number of ways to choose the vertex stereochemistry is investigated (equations 7 and 8). Figure S45 illustrates the effect of each symmetry element on picking a particular vertex stereochemistry. Element E will allow for all vertices to be picked independently ($|X^E|_V = 2^5$). The C_5 rotations fix all vertices when one is chosen ($|X^{C_5}|_V = |X^{C_5^2}|_V = 2^1$). The C_2' rotations allow for three vertices to be picked independently ($|X^{C_2'}|_V = 2^3$). It is important to note that upon reflection or inversion, a Λ metal complex is transformed into a Δ metal complex and vice versa. The remaining symmetry elements

in point group D_{5h} involve a mirror plane through the middle of one or more vertices, which means it is not possible to pick any combination where the symmetry is honoured and all these terms will cancel out ($|X^{\sigma_h}|_V = |X^{S_5}|_V = |X^{S_5^3}|_V = |X^{\sigma_v}|_V = 0$). Following this analysis, we conclude that there are four unique ways to choose the vertices and that all combinations are chiral (equation 3).

$$\left|\frac{X}{D_5}\right|_V = \frac{1}{10}(2^5 + 2 \cdot 2^1 + 2 \cdot 2^1 + 5 \cdot 2^3) = 8 \quad \text{(Eq. 7)}$$

$$\left|\frac{X}{D_{5h}}\right|_V = \frac{1}{20}(2^5 + 2 \cdot 2^1 + 2 \cdot 2^1 + 5 \cdot 2^3 + 0 + 2 \cdot 0 + 2 \cdot 0 + 5 \cdot 0) = 4 \quad \text{(Eq. 8)}$$

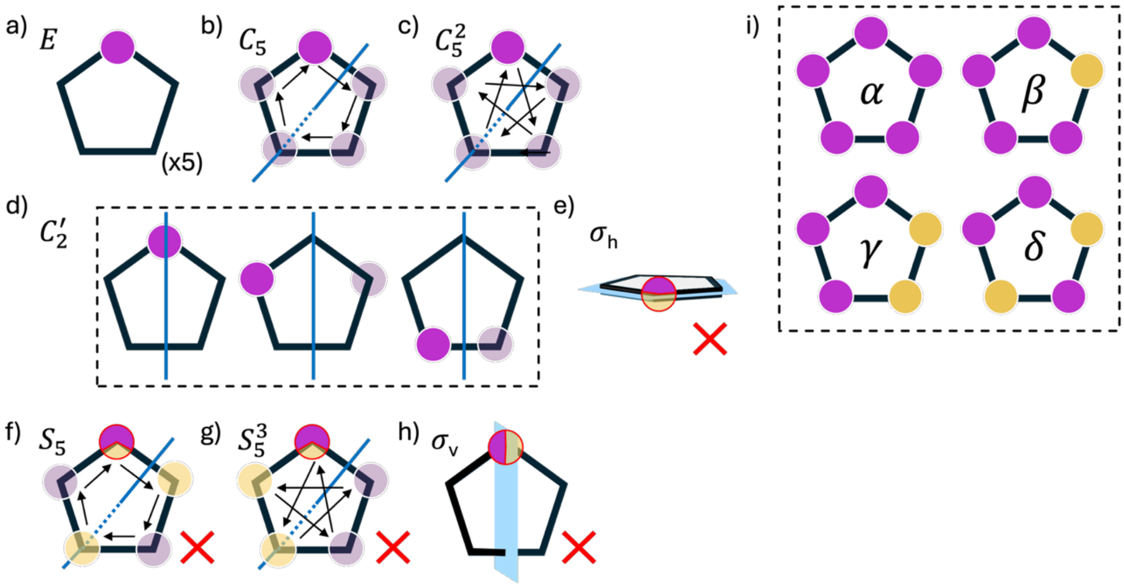

Figure S45. Description of the number of vertices that are fixed by a symmetry element (faded purple or yellow circle) upon picking the orientation of one face (purple circle). A partially purple/yellow circle and red cross are shown when a vertex choice is not symmetric with that symmetry element. The (x5) note is added when all five vertices can be picked independently from each other, resulting in a power-of-5 term in the equation.

Enumeration of distinct pentagonal sandwiches

The information used for the separate calculations for ligand- and vertex-arrangements can be combined (equations 9 and 10).

$$\left|\frac{X}{D_5}\right|_{V,F} = \frac{1}{10}(5^2 \cdot 2^5 + 2 \cdot 0 \cdot 2^1 + 2 \cdot 0 \cdot 2^1 + 5 \cdot 5^1 \cdot 2^3) = 100 \quad \text{(Eq. 9)}$$

$$\left|\frac{X}{D_{5h}}\right|_{V,F} = \frac{1}{20}(10 \cdot 100 + 5^1 \cdot 0 + 2 \cdot 0 \cdot 0 + 2 \cdot 0 \cdot 0 + 5 \cdot 1^2 \cdot 0) = 50 \quad \text{(Eq. 10)}$$

The total number of unique diastereomers (excluding enantiomers), which is calculated using point group D_{5h}, is exactly half that of the total number of diastereomers (including enantiomers), which is calculated with point group D_5 (equation 11). All combinations are chiral and the total number of diastereomers will be doubled when including these extra enantiomers. Intuitively this makes sense, as none of these pentagonal sandwich structures can contain mirror planes. All mirror planes cross at least one vertex, which cannot be honoured as one vertex cannot be Λ and Δ at the same time.

$$\left|\frac{X}{D_5}\right|_{V,F} = 2\left|\frac{X}{D_{5h}}\right|_{V,F} \quad \text{(Eq. 11)}$$

In conclusion, Burnside's Lemma shows that there are 50 unique diastereomers for the pentagonal $Li_5\mathbf{L}_2$ sandwiches. Note that here we calculated the theoretical number of diastereomers assuming that the two pentagon faces are perfectly overlapping each other. The DFT-optimized structure obtained (Section 7.4) has two pyrrole ring that are not perfectly overlapping. Hence the actual number of diastereomers is larger than 50.

7.2 Investigation of the Li-coordination environments

Four different potential geometries for the $Li_5\mathbf{L}_2$ sandwich complex **1** were modelled using the MM3 forcefield in Scigress.[54] By explicitly drawing the various possible Li–N connections, different possible coordination environments around the Li atoms were explored. These four models were used as input structures for further calculations in order to investigate the most feasible coordination environment.

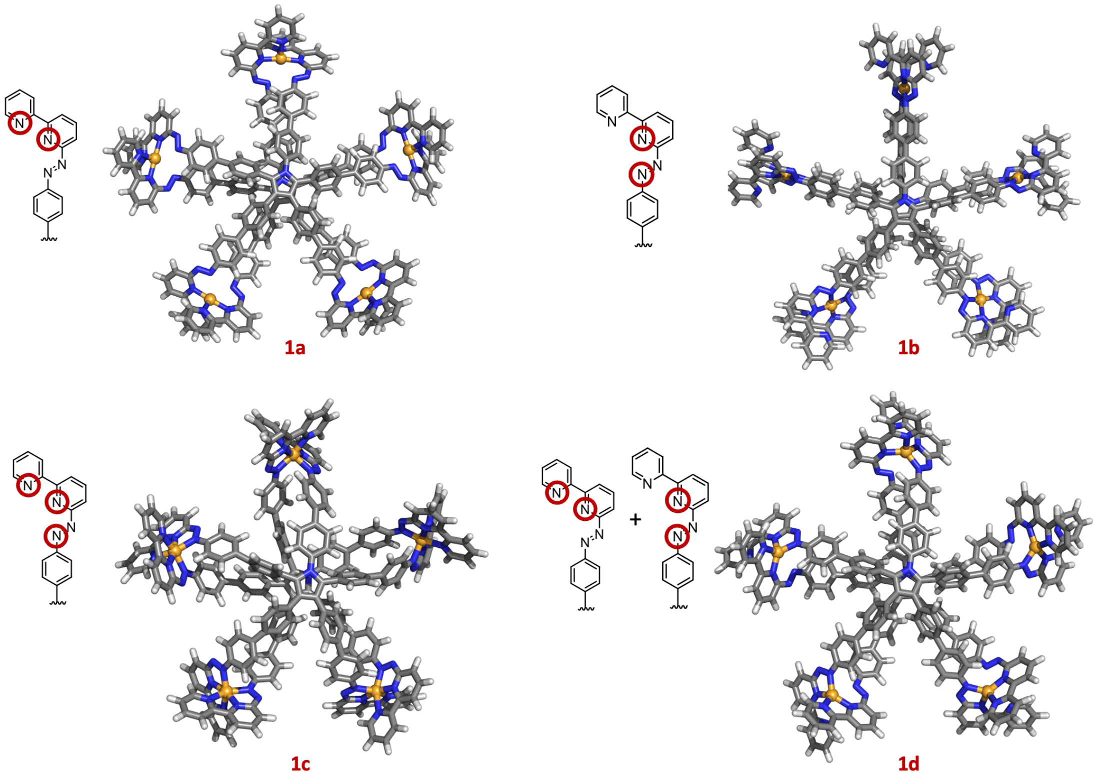

Figure S46. Four structures modelled using the MM3 force field in Scigress[54] that were made by connecting the Li atoms to the N atoms in different ways. Two nitrogen from bipyridine groups on each arm were connected to Li^+ for 1a. One nitrogen from a bipyridine group and one nitrogen from an azo group on each arm were connected to Li^+ atoms for 1b. Two nitrogen atoms from bipyridine groups and one nitrogen from an azo linkage on each arm were connected to Li^+ for **1c**. **1d** is the mixed combination from **1a** and **1b**, where one ligand coordinates to the Li^+, as in **1a,** and the other ligand coordinates as in **1b**.

Further semi-empirical quantum mechanical calculations were carried out on the four models using the OPTIM program,[68] which contains a variety of geometry optimization tools for locating stationary points on potential energy surfaces and calculating reaction pathways. Geometry optimizations were performed on structures **1a**, **1b**, **1c** and **1d** using the OPTIM interface to the xTB program[69] which was employed to calculate the energy and gradient at the GFN2-xTB level of theory.[55–57] For each calculation the overall charge was +5, the accuracy parameter was set to 0.01, and calculations were performed in the gas phase. In all four cases a well-converged structure was obtained. Table S2 shows the resulting total energies in kcal/mol, relative to the lowest energy diastereomer **1a**. The strong similarity of structures **1a** and **1d** (Figure S47b), and their almost identical total energies, indicates that the same minima were found starting from two different input structures.

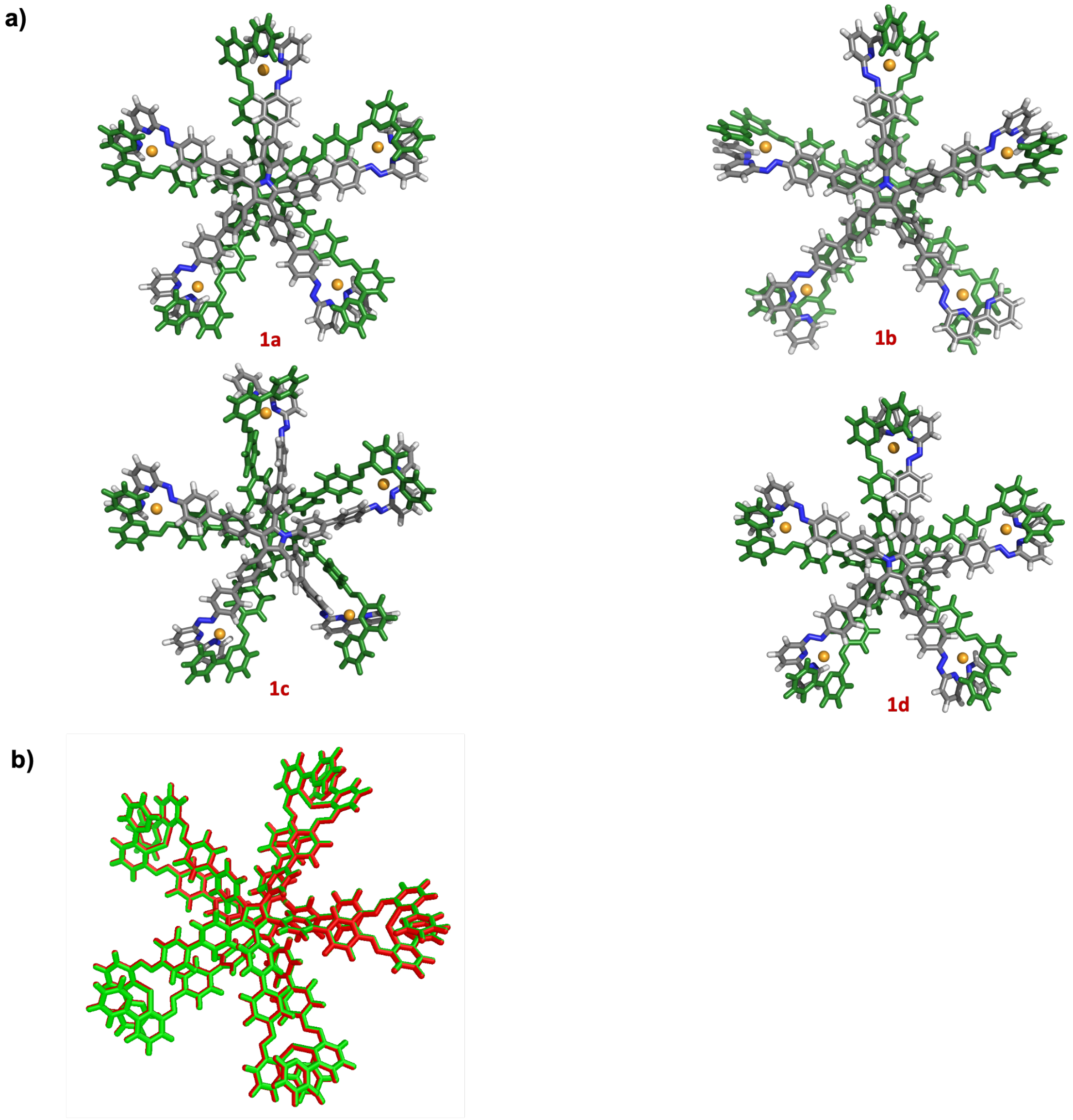

Figure S47. a) GFN2-xTB optimized structure for **1a**-**1d**. For each of the four structures, one ligand **L** is highlighted in green. The MM3 optimized geometries (Figure S46) were used as input structures for these calculations. **b)** Comparison of the GFN2-xTB optimized structures of **1a** (red) and **1d** (green), which converged to the same minimum.

Structure	1a	1b	1c	1d
$E_{GFN2\text{-}xTB}$	0.00	16.63	7.47	0.00

Table S2. Comparison of the energies in kcal/mol for **1a-1d** at the GFN2-xTB level of theory.

To explore the configuration space further we employed basin-hopping global optimization[70,71] using the GMIN program[72] via the interface to XTB. Here we used the GFN-FF level of theory for the global minimum search[73] followed by relaxation at the GFN2-xTB level, again using the OPTIM interface to XTB. The GFN2-xTB optimized structures shown in Figure S47 were used as input. These searches did not locate any physically relevant lower energy minima, suggesting that the candidates discussed above provide a useful basis for our analysis.

The energy level with different vertex arrangements was investigated next. We changed the handiness of the Li center at one arm. As shown in Figure 48, **1a_II** , which has four Δ vertices and one Λ vertex, is higher in energy than **1a**. Hence, the pentagonal sandwich with all identical vertex arrangements, and therefore higher symmetry, is expected to be favored.

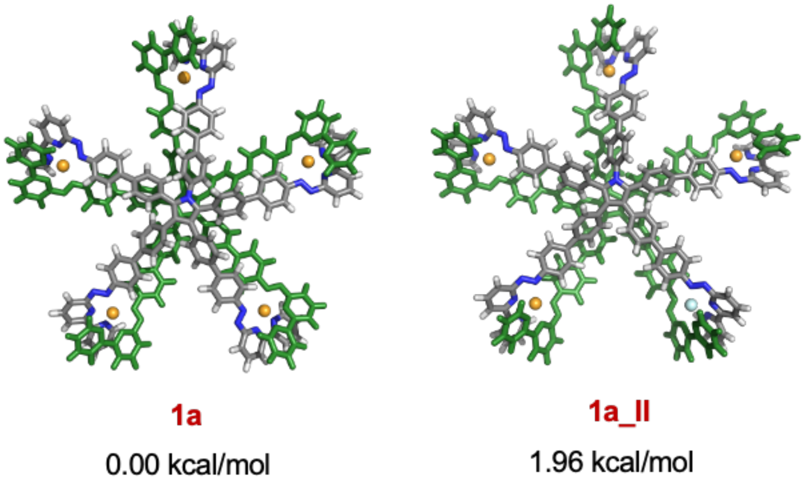

Figure S48. GFN2-xTB optimized structure for **1a_ II** adopting different vertex arrangements. Four Li atoms have *fac*-Δ stereochemistry (yellow) and one has *fac*-Δ (cyan).

7.3 Investigation of the relative pyrrole-nitrogen positions

Configuration **1a** was the lowest energy conformer located in the above searches. Further (semi-empirical) quantum mechanical calculations were considered to investigate the most feasible relative positioning of the pyrrole-N atoms. There are five potential relative orientations of the pyrrole-N atoms, leading to five diastereomers with similar Li-coordination environments. Geometry optimizations at the GFN2-xTB level were

performed on the four diastereomers of **1a** (**1a_*β*-1a_*γ*′**). In each case, the relative positions of the pyrrole-nitrogens were adjusted by moving one pyrrole N-atom around the ring relative to the other pyrrole N-atom, starting from structure **1a_*α***. This construction was performed using the molecular editor Avogadro2. As in the last section, an overall charge of +5 was employed, the accuracy parameter was set to 0.01, and calculations were performed in the gas phase. The calculation resulted into well-converged minima. The resulting energies, relative to **1a_*α***, are reported in kcal/mol in Table S3.

The GFN2-xTB optimized structures were further optimized using Density Functional Theory (DFT). These calculations employed the r^2SCAN-3c[58] method using the ORCA program.[74] First, the normal self-consistent field (NormalSCF) convergence criterion was used, followed by geometry optimizations with the TightSCF setting. A charge of +5 and a spin multiplicity of 1 were employed and calculations were performed in the gas-phase using the RI-J approximation via the def2/J keyword. Structure **1a_*γ*′** was found to have the lowest energy at both levels of theory for this set of five conformations.

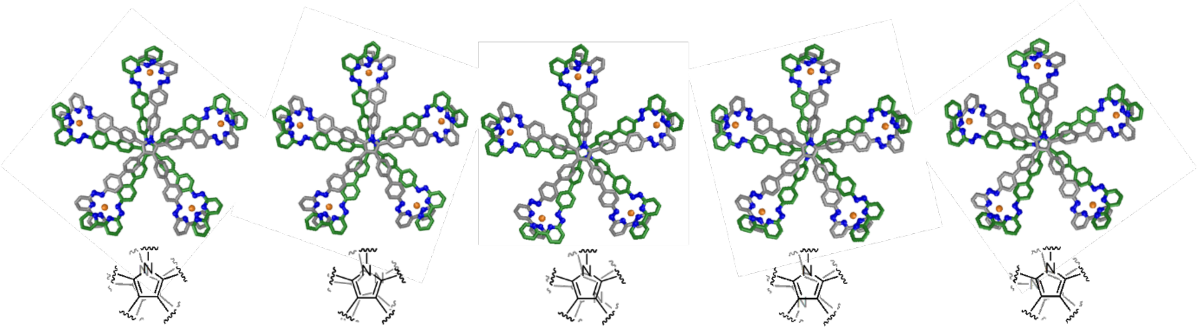

Figure S49. r^2SCAN-3c optimized structures for **1a_*α*** adopting five different relative nitrogen orientations. Hydrogens are omitted for clarity.

**Structure**	**1a_*α***	**1a_*β***	**1a_*γ***	**1a_*γ*′**	**1a_*β*′**
$E_{GFN2\text{-}xTB}$	0.00	-0.11	-0.53	-0.54	-0.41
$E_{r2SCAN\text{-}3c}$	0.00	-0.37	-2.59	-6.29	-6.19

Table. S3. Comparison of the energies in kcal/mol for **1a** adopting different nitrogen orientations. For both methods, **1a_*γ*′** was found to be lowest in energy out of these five geometries.

7.4 Investigation of relative orientations of the phenyl rings

Additional searches for the most favorable structure were conducted by slightly adjusting the orientations of the phenyl rings to look for lower energy minima. Here we were checking for minima that could have been missed in the global minimum searches with GMIN at the GFN-FF level, which do not take into account non-covalent interactions, such as π-π stacking. Following geometry optimizations at the r^{2}SCAN-3c starting from these new perturbed input structures, five new geometries were identified where two phenyl rings were observed to be stacked at each of the five arms. Energies of these new geometries (**1a'_*α*-1a'_*γ'***) were calculated relative to the energy of **1a_*α*** and can be found in Fig. S49. For each of the five diastereomers, the new geometry with the stacked phenyl orientations was found to be lower in energy than the original conformation (See Figure S50 and Table S4). The **1a'_*γ'*** diastereomer was the lowest in energy at this level of theory.

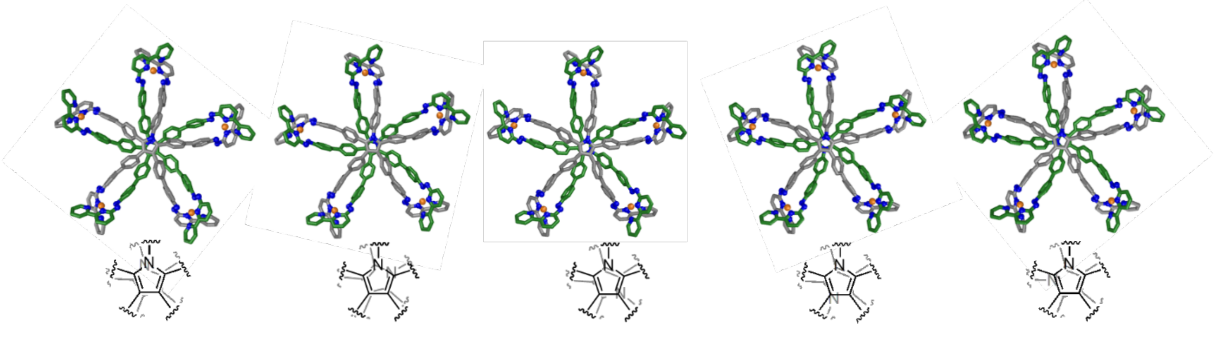

Figure S50. r^{2}SCAN-3c optimized structures for **1a'_*α*** adopting different nitrogen orientation and their energy comparison. Hydrogens are omitted for clarity.

Structure	1a'_*α*	1a'_*β*	1a'_*γ*	1a'_*γ'*	1a'_*β'*
$E_{r2SCAN-3c}$	-7.95	-7.63	-7.94	-8.18	-8.14

Table. S4. Comparison of the energies in kcal/mol for **1a'_*α*** adopting different nitrogen orientations. **1a'_*γ'*** was the lowest energy structure at this level of theory.

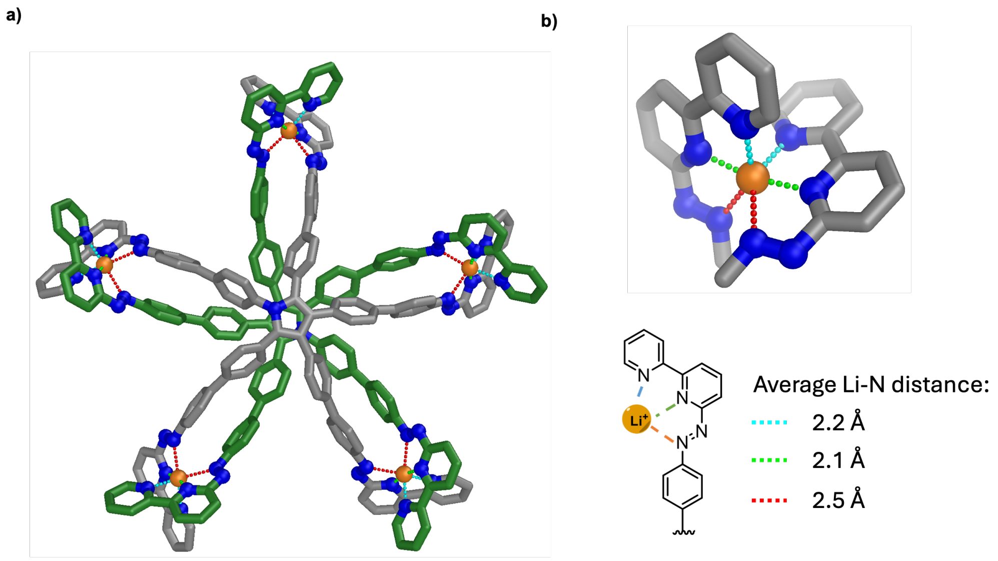

Figure S51. a) The structure for **1a'_**γ'. Hydrogens are omitted for clarity. **b)** Illustration of the Li-coordination environment found at each of the five arms in **1a'_**γ' and the measured averaged Li–N distances.

8. References

64. Li, F.; Yang, B.; Miller, M. J.; Zajicek, J.; Noll, B. C.; Möllmann, U.; Dahse, H.; A. Miller P. A. Iminonitroso Diels−Alder reactions for efficient derivatization and functionalization of complex diene-containing natural products. *Org. Lett.* **2007***, 9*, 2923–2926.

65. Miller, C. C. The Stokes-Einstein law for diffusion in solution. *Proc. R. Soc. Lond. A* **1924**, *106*, 724–749.

66. Schaapkens, X.; Bobylev, E. O.; Reek, J. N. H., Mooibroek, T. J. A $[Pd_2L_4]^{4+}$ cage complex for n-octyl-β-D-glycoside recognition. *Org. Biomol. Chem.* **2020**, *18*, 4734–4738.

67. William, B. *Theory of Groups of Finite Order.* Project Gutenberg, 2012, https://www.gutenberg.org/ebooks/40395 .

68. OPTIM: A program for geometry optimisation and pathway calculations. http://www-wales.ch.cam.ac.uk/software.html.

69. Wesolowski, P. A.; Wales, D. J.; Pracht, P. Multilevel framework for analysis of

protein folding involving disulfide bond formation. *J. Phys. Chem. B* **2024**, *128*, 3145–3156.

70. Wales, D. J.; Doye, J. P. K. Global optimization by basin-hopping and the lowest energy structures of Lennard-Jones clusters containing up to 110 atoms. *J. Phys. Chem. A* **1997**, *101*, 5111–5116.

71. Li, Z.; Scheraga, H. A. Monte Carlo-minimization approach to the multiple-minima problem in protein folding. *Proc. Nat. Acad. Sci.* **1987**, *84*, 6611–6615.

72. GMIN: A program for Basin-Hopping global optimisation, Basin-Sampling, and Parallel Tempering. http://www-wales.ch.cam.ac.uk/software.html.

73. Spicher, S.; Grimme, S. Robust atomistic modeling of materials, organometallic, and biochemical systems. *Angew. Chem. Int. Ed.* **2020**, *59*, 15665–15673.

74. Neese, F.; Wennmohs, F.; Becker, U.; Riplinger, C. The ORCA quantum chemistry program package. *J. Chem. Phys.* **2020**, *152*, 224108.
